# Targeting DNA junction sites by bis-intercalators induces topological changes with potent antitumor effects

**DOI:** 10.1093/nar/gkae643

**Published:** 2024-07-22

**Authors:** Shih-Chun Huang, Chia-Wei Chen, Roshan Satange, Chang-Chih Hsieh, Chih-Chun Chang, Shun-Ching Wang, Chi-Li Peng, Tai-Lin Chen, Ming-Hsi Chiang, Yih-Chern Horng, Ming-Hon Hou

**Affiliations:** Doctoral Program in Medical Biotechnology, National Chung Hsing University, Taichung 402, Taiwan; Graduate Institute of Genomics and Bioinformatics, National Chung Hsing University, Taichung 402, Taiwan; Department of Chemistry, National Changhua University of Education, Changhua 50058, Taiwan; Graduate Institute of Genomics and Bioinformatics, National Chung Hsing University, Taichung 402, Taiwan; Institute of Chemistry, Academia Sinica, Taipei 11528, Taiwan; Graduate Institute of Biotechnology, National Chung Hsing University, Taichung 402, Taiwan; Doctoral Program in Medical Biotechnology, National Chung Hsing University, Taichung 402, Taiwan; Graduate Institute of Genomics and Bioinformatics, National Chung Hsing University, Taichung 402, Taiwan; Graduate Institute of Genomics and Bioinformatics, National Chung Hsing University, Taichung 402, Taiwan; Post Baccalaureate Medicine, School of Medicine, National Chung Hsing University, Taichung 402, Taiwan; Institute of Chemistry, Academia Sinica, Taipei 11528, Taiwan; Department of Chemistry, National Changhua University of Education, Changhua 50058, Taiwan; Doctoral Program in Medical Biotechnology, National Chung Hsing University, Taichung 402, Taiwan; Graduate Institute of Genomics and Bioinformatics, National Chung Hsing University, Taichung 402, Taiwan; Graduate Institute of Biotechnology, National Chung Hsing University, Taichung 402, Taiwan; Biotechnology Center, National Chung Hsing University, Taichung 402, Taiwan

## Abstract

Targeting inter-duplex junctions in catenated DNA with bidirectional bis-intercalators is a potential strategy for enhancing anticancer effects. In this study, we used d(CGTATACG)_2_, which forms a tetraplex base-pair junction that resembles the DNA–DNA contact structure, as a model target for two alkyl-linked diaminoacridine bis-intercalators, DA4 and DA5. Cross-linking of the junction site by the bis-intercalators induced substantial structural changes in the DNA, transforming it from a B-form helical end-to-end junction to an over-wounded side-by-side inter-duplex conformation with A-DNA characteristics and curvature. These structural perturbations facilitated the angled intercalation of DA4 and DA5 with propeller geometry into two adjacent duplexes. The addition of a single carbon to the DA5 linker caused a bend that aligned its chromophores with CpG sites, enabling continuous stacking and specific water-mediated interactions at the inter-duplex contacts. Furthermore, we have shown that the different topological changes induced by DA4 and DA5 lead to the inhibition of topoisomerase 2 activities, which may account for their antitumor effects. Thus, this study lays the foundations for bis-intercalators targeting biologically relevant DNA-DNA contact structures for anticancer drug development.

## Graphical Abstract

**Graphical Abstract:**
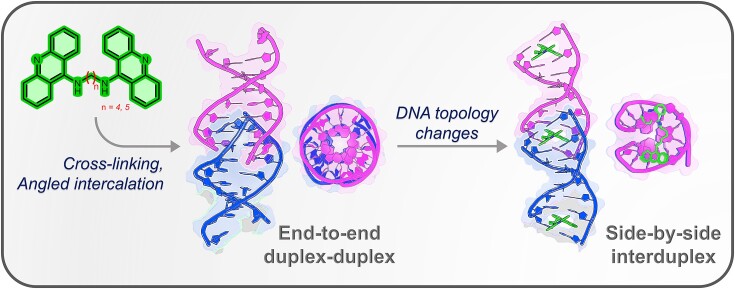

## Introduction

**Figure 1.**
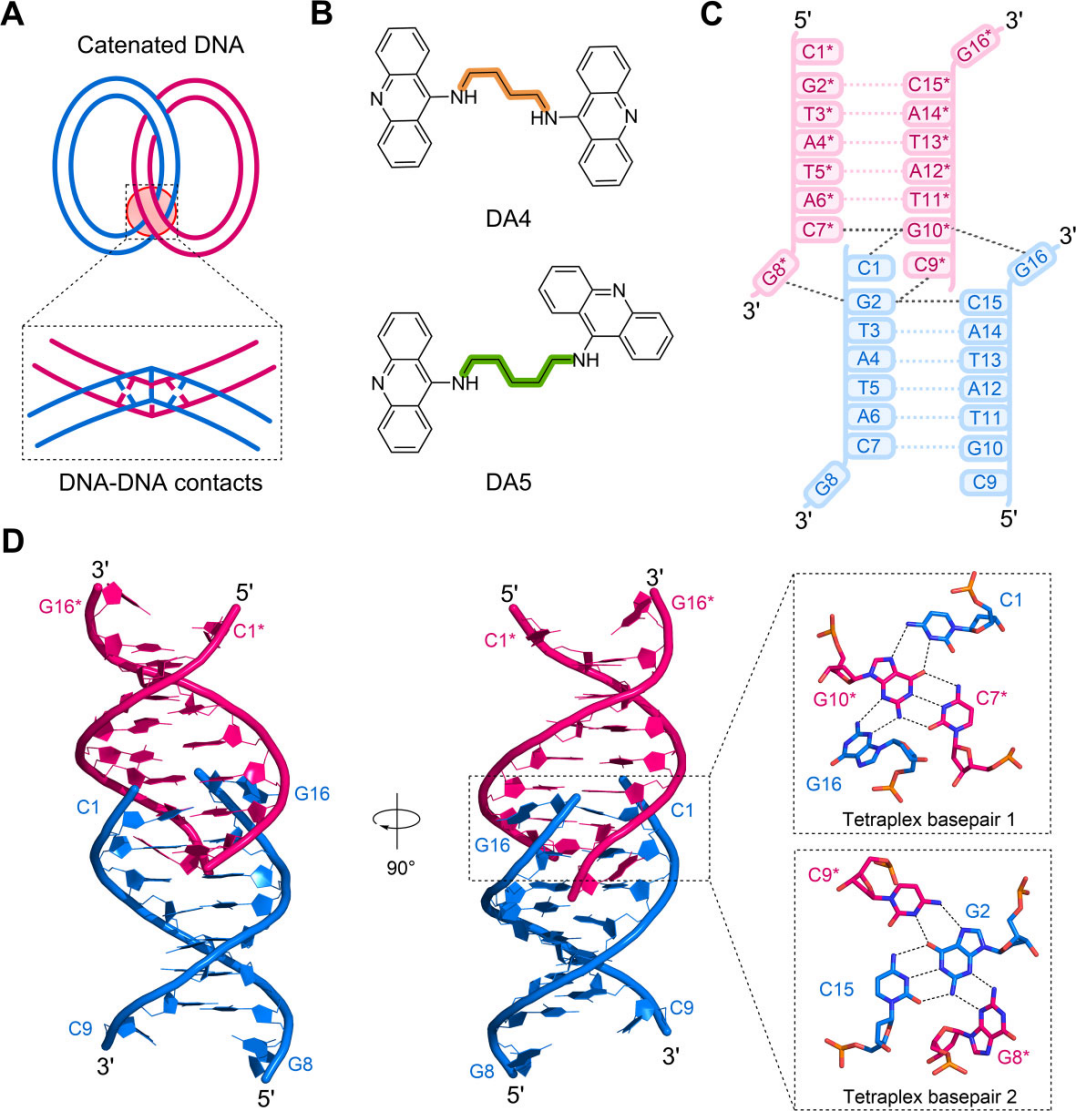
Structural features of the d(CGTATACG)_2_ DNA. (**A**) Schematic representation of the DNA-DNA contact structures formed in catenated DNA. (**B**) Chemical structures of the alkyl-linked diaminoacridine bis-intercalators DA4 (Top) and DA5 (Bottom) used in this study. (**C**) Schematic representation and numbering scheme of the crystal structure of d(CGTATACG)_2_ indicating the residues involved in junction formation. One DNA duplex is shown in blue, while the adjacent symmetry-related duplex is colored pink. Asterisks (*) represent residues in adjacent duplex in crystal symmetry. (**D**) Crystal structure assembly of continuous duplexes in d(CGTATACG)_2_ DNA forming an end-to-end junction structure. Two layered tetraplex base pairings at the junction site are shown in an enlarged view.

DNA is densely packed into the chromatin environment within cells. Despite this tight packing, the double-helical DNA remains accessible to various proteins involved in cellular processes through large-scale structural modulations in DNA assemblies (1,2). These processes often result in neighboring DNA–DNA contact in cells. For example, duplex–duplex catenated structures are formed at sites where two replication forks collide during replication (3–5). The crossover sites in catenated DNA could resulted in specific interactions of the bases within the inter-duplex structures (Figure 1A) (6,7). Since, topoisomerases facilitate the decatenation of these structures, the crossover structures can serve as a target site for the binding of these enzymes (8–12). In addition, studies have shown that the CG-rich sequences at DNA branch point can lead to the formation of various junction structures (13,14). Targeting DNA–DNA contact sites with bidirectional linkers containing bis-intercalators could be an alternative strategy to inhibit topoisomerase functions (15–17). In rapidly dividing cancer cells, the inhibition of topoisomerase activity can impede cell proliferation, thereby achieving a therapeutic effect for cancer treatment (18–20).

DNA bis-intercalators exhibit higher binding affinity and reduced dissociation rates, enhancing target specificity (21–23). These bis-intercalators are categorized as intra-duplex and inter-duplex bis-intercalators based on the intercalation capabilities of their chromophores. Intra-duplex bis-intercalators insert into a single DNA molecule, whereas inter-duplex bis-intercalators possess the unique ability to insert their chromophores into the base pairs of two distinct duplexes simultaneously (24–26). This unique ability enables them to bridge and non-covalently cross-link two duplexes, influencing biological processes (27). To facilitate DNA cross-linking through bis-intercalation, the development of bis-intercalators with less flexible linkers has been proposed (28–30). Furthermore, bis-intercalators with short and moderately flexible linkers have been suggested for incorporation at DNA junction sites (31–33). Despite their significance as chemotherapeutic agents, the detailed binding mechanisms and biological consequences of these agents remain to be elucidated.

This study determined the crystal structure of a self-complementary DNA, d(CGTATACG)_2_, which displayed a unique duplex–duplex junction. The overall features of the current structure exhibited B-DNA-like characteristics in the central helical region; however, the terminal -CG- base pairs contributed to the formation of two tetraplex base pairs. Detailed analysis revealed that the structure resembles duplex–duplex contacts in catenated DNA and might serve as a hotspot to accommodate the external ligand into the two neighboring duplexes at the tetraplex interface. To explore the possibility of targeting inter-DNA duplex structures, we synthesized two acridine-based derivatives, DA4 and DA5, by coupling 9-aminoacridine moieties with hydrocarbon chains of four and five atoms, respectively (Figure 1B). We determined the crystal structures of DA4 and DA5 in complex with d(CGTATACG)_2_. The angled intercalation of diacridines induces extensive structural changes in the DNA, resulting in a side-by-side duplex–duplex cross-linking complex. Our structural analysis of these complexes revealed that DA5, with its more flexible linker and increased DNA interactions exhibits a stronger TOP2 inhibitory and anticancer activities than DA4. Detailed structure-based studies on the capabilities of bidirectional linker-containing bis-intercalators are crucial for understanding their potential ability to bind to inter-duplex contacts, which may open new avenues for targeting biologically relevant DNA structures.

## Materials and methods

### Chemicals and DNA oligonucleotides

The compounds DA4 and DA5 were synthesized from commercially available analytical grade reagents (34). The detailed synthesis scheme for DA4 and DA5 is given in the supplementary note. DNA oligomers purified by polyacrylamide gel electrophoresis were purchased from MDBio, Inc. (New Taipei City, Taiwan). Oligonucleotide solutions were prepared in double-distilled water (ddH_2_O), followed by heating at 95°C for 5 min and slow cooling (–0.5°C/min) to room temperature to facilitate annealing. The concentration (*c*) of each oligonucleotide sequence was calculated using Beer's law (*A* = ϵ* b* *c*), where the absorbance (*A*) was measured at 260 nm using an ultraviolet-visible spectrophotometer (JASCO International Co. Ltd, Tokyo, Japan) in a quartz cuvette with path length (*b*) = 1 cm. The values of oligomer extinction coefficient (ϵ) were estimated using DNA Calculator software (Molbiotools).

### CD spectroscopy and thermal stability assays

All spectra were recorded at 25°C using a Chirascan™ V100 CD spectrophotometer (v4.8.3.313) equipped with the Pro-Data Software suite (v4.8.3.0) and a quartz cuvette with path length = 1 mm. Oligonucleotide sequences were mixed at a concentration of 20 μM in a buffer containing 20 mM sodium cacodylate (pH 7.3), 100 mM potassium chloride and 5 mM magnesium chloride hexahydrate. The reaction mixture was heated to 95°C for 5 min and then allowed to cool slowly. Various ratios of compounds were incubated with the oligonucleotide samples. Ellipticity spectra for CD were recorded from 300 to 235 nm with a sampling rate of 1 s and were analyzed by curve fitting (35). For the analysis of melting temperature (*T*_m_), 3 μM d(CGTATACG)_2_ oligonucleotides were incubated in the same buffer as the CD experiment with a 1:4 DNA:compound ratio. UV melting curves were obtained by measuring the absorbance at 260 nm from 4°C to 95°C at a rate of 1°C/min in the presence and absence of compounds using a JASCO UV/VIS spectrophotometer. *T*_m_ values were calculated by using the least squares method in Spectra Manager version 2.0 (JASCO International Co. Ltd, Tokyo, Japan).

### Stoichiometry analysis

Job-type titration was used to determine the stoichiometry of DA4 and DA5 binding to the d(CGTATACG)_2_ DNA sequence using CD spectroscopy. The total concentration of DNA and compounds was set at 100 μM. Samples were prepared in a buffer containing 50 mM sodium cacodylate (pH 7.3) and 5 mM magnesium chloride hexahydrate by heating and cooling as described earlier, and then incubated at 4°C overnight. The CD spectra of the samples were measured at 25°C. The data corresponding to the titration of DA4 and DA5 with DNA, as intensity changes at 272 nm, were fitted using non-linear regression (curve fitting) in GraphPad Prism v9.0.

### Crystallization

All crystallization experiments were conducted using the sitting-drop vapor diffusion method. Crystals of d(CGTATACG)_2_ DNA alone were grown by mixing 1.0 mM oligonucleotide solution with a mother liquor containing 50 mM bis–tris (pH 7.0), 20 mM NaCl, 10 mM CaCl_2_, 80 mM KCl, 7 mM MnCl_2_·4H_2_O and 10% (v/v) PEG2000 and equilibration against 500 μl of 50% PEG2000. Crystals of DA4–DNA complex were obtained by combining 0.5 mM d(CGTATACG)_2_ oligonucleotides with 1 mM DA4 in a buffer containing 200 mM KCl, 6 mM MnCl_2_·4H_2_O, 50 mM MES (pH 6.3), and 10% PEG400 and equilibration against 200 μl of the mother liquor. Small, yellow, needle-shaped crystals formed within a few weeks at an incubation temperature of 20°C. For DA5–DNA crystals, 0.3 mM d(CGTATACG)_2_ oligonucleotides were combined with a solution containing 25 mM bis–tris (pH 7.0), 50 mM NaCl, 10 mM MgCl_2_·6H_2_O and 15% (v/v) 2-methyl-2,4-pentanediol in a 1:1 DNA:DA5 ratio and equilibrated against 200 μl of mother liquor. Light yellow crystals appeared after approximately one month at 20°C.

### X-ray data collection, phasing and structure refinement

X-ray diffraction data were collected at the NSRRC, Taiwan. Data reduction, processing, integration and scaling were performed using the HKL-2000 software package (36). PHENIX (v1.18.2–3874) was used for phase determination of d(CGTATACG)_2_ DNA-alone and the DA4–DNA and DA5–DNA complexes by molecular replacement (phaser MR) in the *P*4_1_2_1_2, *C*222_1_ and *P*2_1_ space groups, respectively (37). The crystallographic model of the DNA decamer (PDB ID-237D) and a model of a typical B-form DNA duplex created with Discovery Studio 2020 Client (v20.1.0.19295) served as templates for the initial phasing of the d(CGTATACG)_2_ DNA-alone duplex structure. This structure then facilitated phase determination for the DA4–DNA and DA5–DNA complexes. Structural refinement was conducted using phenix.refine package in PHENIX (v1.18.2–3874) and WinCoot (v0.8.9.2) (38). Detailed crystallographic and final refinement statistics are provided in Supplementary Table S1. Final 2mF_o_-DF_c_ maximum likelihood-weighted Fourier electron density maps were generated with fast Fourier transform in CCP4i and PyMOL, which was used to create graphical representations of the refined structures. DNA structural parameters, including helical parameters, torsion angles, and sugar puckers, were calculated using the Curves+ online web server (39). Local base-pair parameters and local base-pair step parameters were determined using the Web-3DNA server (40). The values of these parameters for each complex structure in this study are listed in Supplementary Tables S2 and S3.

### Human topoisomerase II (TOP2) activity assay

For the TOP2 activity inhibition assay, kDNA served as the substrate. Relaxed circular DNA and linear kDNA were used as markers. The reaction mixture was prepared in freshly made 5X complete reaction buffer containing 0.5 M Tris–HCl (pH 8.0), 1.5 M NaCl, 100 mM MgCl_2_, 5 mM dithiothreitol, 300 μg/ml bovine serum albumin, and 20 mM ATP. To this buffer, 100 ng of kDNA, test compounds at six concentrations (1, 2, 5, 10, 15 and 20 μM) and two units of TOP2 were added to a final volume of 20 μl. Initially, the mixture of kDNA and compounds was incubated at 4°C for 45 min. Subsequently, TOP2 was introduced, and the reaction was incubated at 37°C for an additional 45 min. The reaction was terminated with a 5× stop buffer. Doxorubicin was served as control. At the end of the reaction, samples were electrophoresed on a 1% agarose gel in tris-acetate-ethylenediaminetetracetic acid (TAE) buffer with ethidium bromide (0.5 μg/ml). Post-electrophoresis, the gel was destained in water for 20 min, and imaged using Uni-photo (EZ-lab).

### DNA polymerase I activity assay

For the polymerase activity assay, *E. coli* DNA polymerase I (POL I), a DNA template containing 5′-TCCCCGCGCGCCCGAGCGCGCCTCCGCCCTTGCCCGCCCCCTGACGCTGCCTCA-3′, and a primer sequence (5′-TGAGGCAGCGTCAGGGGGCG-3′) from hypoxia-inducible factor 1-alpha promoter region were used owing to the presence of multiple CG sites. Briefly, the template-primer mixture was heated at 95°C for 5 min in 2 μl of 10× reaction buffer (500 mM Tris–HCl, pH 7.5; 100 mM MgCl_2_; 10 mM DTT), 1 μl of a dNTP mixture, and ddH_2_O and then cooled on ice for annealing. The test compounds were added at three concentrations (10, 25 and 50 μM) along with POL I (10 units) to a final volume of 20 μl. Actinomycin D (ActD) served as a control. The template primer and test compound were incubated at 4°C for 45 min, followed by the incubation of the template primer, compound, and POL I at 37°C for 20 min. Subsequently, 7.5 μl of the sample was combined with 1.5 μl of 6× DNA loading dye and heated at 95°C for 5 min. Electrophoresis was conducted on a 12% native gel containing tris-borate-ethylenediaminetetraacetic acid (TBE) buffer. The gel was stained with SYBR Gold (diluted 1:20 000 in 1× TBE buffer) for 15 min, destained with water for 20 min, and imaged using a DigiGel system (DGIS-10c).

### Cell culture and cell viability assay

SW620 and A549 cells were cultured in Dulbecco's modified Eagle's medium (DMEM) supplemented with 10% fetal bovine serum and 1% antibiotic–antimycotic solution and maintained at 37°C in a 5% CO_2_ incubator. To assess cell viability, cells were seeded into 96-well plates at 5 × 10^3^ cells per well and incubated overnight at 37°C. Diacridine compounds were prepared in DMEM and added to each well at various concentrations. Cells were incubated with the compound or DMSO as the control for 48 h. Post-incubation, the anticancer efficiency of the compounds was evaluated using the 3-(4,5-dimethylthiazol-2-yl)-2,5-diphenyltetrazolium bromide (MTT) assay. After removing the original culture medium, 100 μl of MTT solution was added to each well. After incubation with MTT, the absorbance of the treated cells at 570 nm was measured using an enzyme-linked immunosorbent assay (ELISA) reader to determine cell viability. IC_50_ values, which represent the concentration of compounds required to inhibit 50% of cancer cells, were determined from more than three independent experiments.

### Cell cycle assay

Cells were seeded in 6-well plates at a density of 1 × 10^5^ cells per well and treated with 25 μM diacridines for 12 h. After treatment, cells were fixed with 70% EtOH at –20°C. The cells were then stained with a PI solution containing 150 μl of phosphate-buffered saline, PI at a ratio of 500:1, and RNase A at a ratio of 200:1, and 0.05% Tween-20 was added to each well and incubated at room temperature for 15–30 min. DNA content was assessed by analyzing the cells with an Accuri™ C5 cytometer, and data were processed using FlowJo V10 software. Experiments were conducted at least three times to ensure the reliability and reproducibility of the results.

### Apoptosis assay

Cells were cultured in 6-well plates at a density of 2 × 10^5^ cells per well and incubated overnight. Subsequently, the cells were treated with media containing 25 μM compounds for 24 h. After treatment, cells were stained with an Annexin V-fluorescein isothiocyanate (FITC)/PI apoptosis detection kit (Thermo Fisher Scientific) for 15 min, with untreated cells serving as the control group. Early and late apoptotic cells were identified by flow cytometry. Analysis was performed using an Accuri™ C5 cytometer to confirm apoptosis induction, and the resulting data were analyzed with the FlowJo V10 software.

### Therapeutic efficacy in the SW620 xenograft model

Animal studies were conducted in accordance with the IACUC guidelines and protocols (IACUC No. 109–024^R^) approved by the Institutional Animal Care and Use Committee at National Chung Hsing University. Female BALB/C/Athymic NCr-nu/nu mice aged 7–8 weeks from the National Laboratory Animal Center, Taiwan, were used in the study. For the experiment, 5 × 10^6^ SW620 cells suspended in culture medium were subcutaneously injected into the right flank of the mice. When the tumor volume reached approximately 100 mm^3^, the mice were randomly assigned to one of the following groups: Control (*n* = 5), DA4 (*n* = 6) and DA5 (*n* = 6). The compounds were prepared in 5 mg/ml bovine serum albumin and administered intraperitoneally. The control group received only the vehicle (5 mg/ml bovine serum albumin), while the treatment groups received a 1.2-mg/kg dose of each compound. The mice were treated with the compounds once a week for 4 weeks. The body weights and tumor volumes of the mice were measured twice weekly. Signs of toxicity were monitored throughout the experiment. The body weight loss threshold of 15% was not exceeded. Tumor volume (TV) was calculated using the formula TV = (*L* × *W*^2^) × 0.5, where *L* and *W* represent the length and width of the tumor, respectively, and were measured using a digital caliper. Mice were euthanized with carbon dioxide, and their tumors and livers were excised for weight determination.

## Results

### Crystal structure of d(CGTATACG)_2_ DNA forms duplex–duplex contact with tetraplex base pairing

To understand the basis for crossover structure formation at two DNA interfaces, we used a d(CGTATACG)_2_ DNA with terminal CG sites flanked by AT-rich sequences as a model and determined its crystal structure in *P*4_1_2_1_2 space group at a resolution of 2.70 Å (Supplementary Figure S1A). The structure assembly of this complex featured symmetry related identical intertwined right-handed duplexes (Supplementary Figure S1B). These duplexes formed a helix–helix junction at the terminal C–G base pairs. To facilitate discussion, the oligonucleotides on one strand were numbered from C1 to G8 and those on the complementary strand were numbered from C9 to G16 in the 5′→3′ direction; however, symmetry-related duplexes were denoted with an asterisk (*) (Figure 1C). Detailed analysis of this structure revealed that the distortion of the terminal C–G base pairs created a cavity that accommodates the ends of another duplex, forming a right-handed helix–helix junction structure with two duplexes in close proximity (Figure 1D). The interface of this junction consisted of a two-layered arrangement of four-stranded base pairs. The first tetraplex base pairing was formed between C7*–G10* Watson–Crick base pairing of one duplex and the terminal bases C1 and G16 of the other duplex. Similarly, the second tetraplex base pairing was formed between the G2–C15 Watson–Crick base pairing of one duplex and the terminal G8* and C9* bases of the other duplex. This feature partially resembles the crossover sites containing DNA–DNA contact structure in catenated DNA (4,7,10). The flexible topology of DNA may serve as a target for the binding of DNA bis-intercalating compounds at the crossover CG sites, mediating DNA duplex–duplex contact structures.

### Angled intercalation of DA4 and DA5 into DNA resulted in inter-duplex cross-linking

Studies have shown that the short linker-containing diacridine compounds have shown a preference for intercalating at CG-rich sites in DNA (41–43). To assess the binding capabilities of DA4 and DA5 to d(CGTATACG)_2_ DNA, we examined their stoichiometry using Job-type titration (Supplementary Figure S2). The Job-type titration revealed a distinct peak at approximately 0.66 (mole fraction of the compound), suggesting a 2:1 stoichiometry of the compounds to DNA (44,45). To understand the structural basis of the binding of DA4 and DA5, we determined the crystal structures of DA4 and DA5 with the d(CGTATACG)_2_ sequence in the *C*222_1_ and *P*2_1_ space groups, respectively, at a resolution of 1.58 Å (Supplementary Figure S3). We designated these structures as DA4–DNA and DA5–DNA complexes. The structure of the DA4–DNA complex features an asymmetric unit with a single duplex intercalated with one DA4 moiety, two Mn^2+^ ions, and 42 water molecules. In contrast, the asymmetric unit of the DA5–DNA complex contains two independent duplexes, each intercalated by a single DA5 moiety and two Mg^2+^ ions, which form octahedral coordination with five water molecules and one phosphate oxygen from the thymine bases.

**Figure 2.**
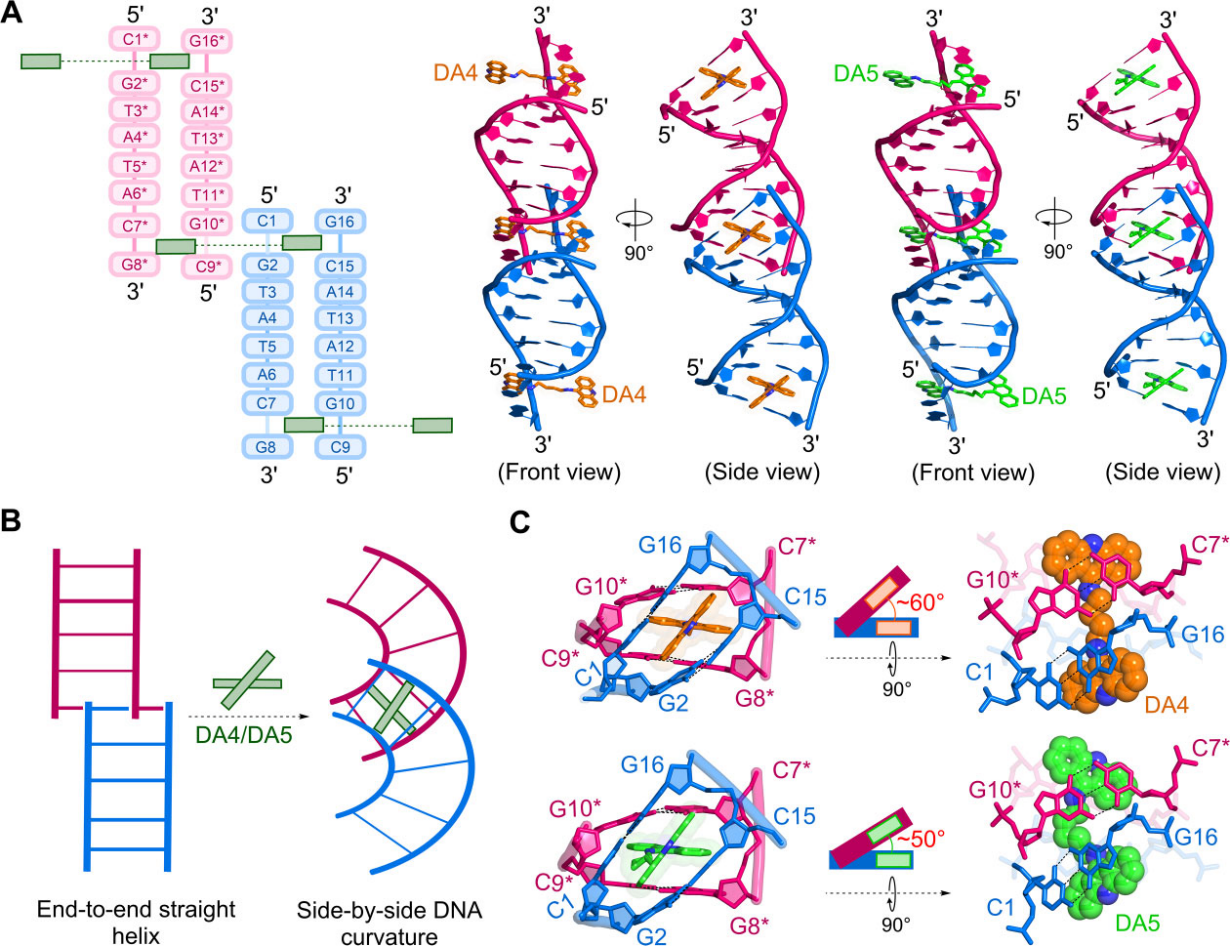
Angled intercalation of DA4 and DA5 into d(CGTATACG)_2_ DNA. (**A**) Schematic representation and numbering scheme of the crystal structures of the two diacridine-DNA complexes. One DNA duplex is shown in blue, whereas the other adjacent duplex is shown in pink. The intercalation of DA4 or DA5 (shown by two green boxes connected by a dashed line) at the terminal CpG sites in each duplex mediates contact between adjacent duplexes by cross-linking. Crystal structure assembly of DA4–DNA and DA5–DNA complexes showing inter-duplex cross-linking of the DNA duplexes. (**B**) Schematic diagram showing topological changes in DNA upon intercalation of DA4 and DA5 at terminal junction sites. (**C**) Magnified view of the intercalation site shows the angled intercalation of DA4 (orange color) and DA5 (green color) at steps C1pG2/C15pG16 in one duplex and C7*pG8*/C9*pG10* in the other adjacent duplex, viewed from the front and top. In DA4, the linker connecting the two acridine moieties is straight, whereas the linker in DA5 has a bent conformation. A single-atom difference in the linker of DA4 and DA5 led to distinct propeller geometries of approximately 60° and 50° in two ligands.

In contrast to the unliganded DNA structure, the crystal assembly of the DA4–DNA and DA5–DNA complexes revealed that the duplexes were juxtaposed near terminal CpG sites (C1pG2 and C7*pG8*, respectively), with DA4 and DA5 bis-intercalating into neighboring duplexes (Figure 2A). Superimposition of individual neighboring duplexes in both DA4–DNA and DA5–DNA structures demonstrated that these duplexes were identical and mediated by crystal packing (Supplementary Figure S4A). However, aligning the upper CpG site (C1–G16/G2–C15) with the lower CpG site (C9*–G8*/G10*–C7*) of adjacent duplexes revealed significant differences at two chromophore insertion sites, suggesting that bidirectional intercalation of DA4 and DA5 had distinct effects on adjacent duplexes (Supplementary Figure S4B). A clear electron density map for both DA4 and DA5 indicated that there was no disorder in modeling the chromophore positions (Supplementary Figure S5). Thus, intercalation of DA4 or DA5 mediated DNA–DNA contacts and cross-linked adjacent duplexes. These observations were consistent with Job-plot titration analyses, which identified two ligand molecules associated with one duplex. Moreover, the current complex structures did not exhibit direct end-to-end stacking interactions between individual duplexes, as observed in the native DNA structure. Instead, the intercalation of DA4 and DA5 induced changes in the DNA topology from an end-to-end straight helix to a side-by-side curvature geometry (Figure 2B). The bidirectional linkers of DA4 and DA5 allowed the intercalation of their two chromophores into the terminal CpG step through the minor groove sides, where the linkers of DA4 and DA5 showed major differences in orientation (Figure 2C): the DA4 linker formed a straight geometry, whereas the DA5 linker bent toward one of the DNA backbones. These complex structures underscored the ability of DA4 and DA5 to induce inter-duplex DNA cross-linking owing to their short and flexible linker properties.

### Linker flexibility of DA5 exhibits strong binding effects on DNA in comparison with the shorter linker of DA4

In both complex structures, each chromophore of DA4 and DA5 was intercalated in parallel with the DNA base pairs; however, a striking difference was observed in the orientation of their linkers. The four-carbon linker of DA4 adopted a relatively straight conformation, in contrast to the five-carbon linker of DA5, which bent toward the DNA backbone. Both DA4 and DA5 linkers were flexible, with sp3-hybridized carbons, and density functional theory calculations indicated that both linkers required only a minor amount of free energy—about 1.00 kcal/mol in solution—for the conversion from a straight to a bent conformation for unbound DA4 or DA5. However, DA5, with its longer hydrocarbon chain, could adopt a greater variety of conformations than DA4. In the current structures, the distance between the two chromophores of DA4 and DA5 was ∼8.5 Å. The difference in linker flexibility allowed the chromophores of DA4 and DA5 to stagger at different angles. In the DA4–DNA complex, the two chromophores formed an angle of ∼60° with the horizontal plane of the acridine ring, whereas in the DA5–DNA complex, the angle between the two acridine chromophores was ∼50°. This flexibility in the DA5 linker resulted in its chromophore aligning in an optimal position to form continuous stacking interactions with DNA base pairs.

**Figure 3.**
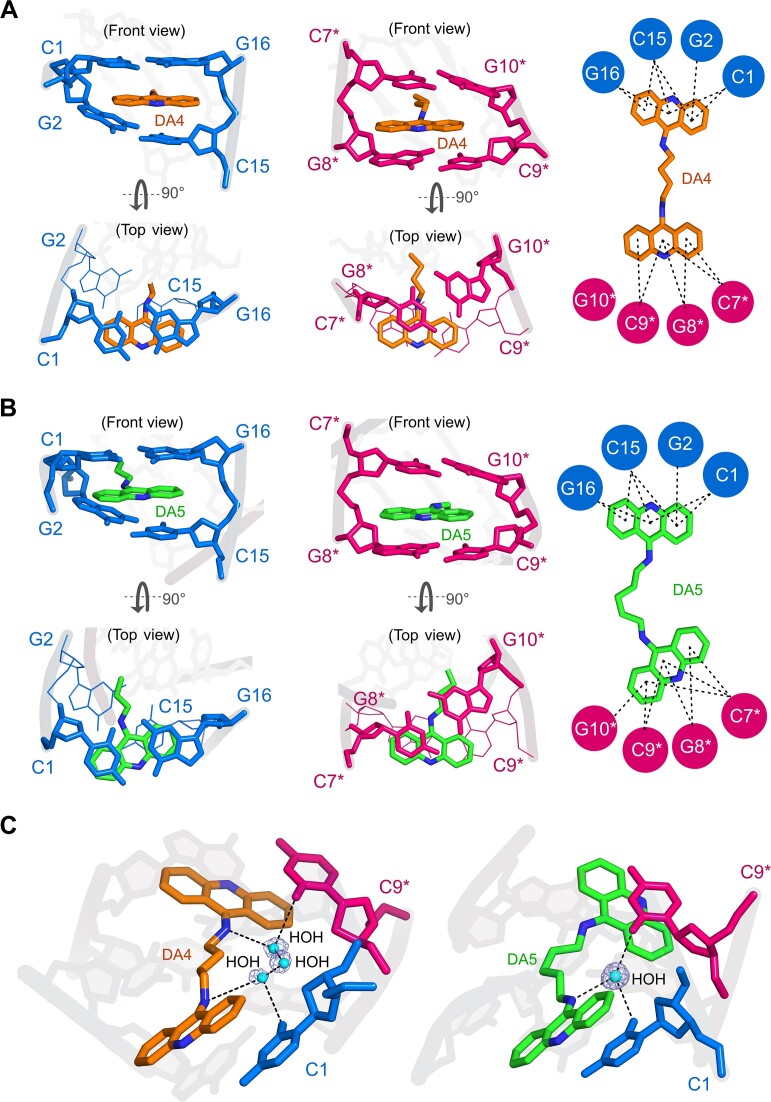
Details of the interactions of DA4 and DA5 with DNA. Overall stacking interactions between (**A**) DA4 and (**B**) DA5 with the terminal CpG sites in each duplex. Intercalation of one chromophore of DA4 (orange stick) and DA5 (green stick) into the C1pG2/C15pG16 steps in one duplex (blue stick representation) and intercalation of the other chromophore of DA4 and DA5 into the adjacent duplex at C7*pG8*/C9*pG10* (pink stick representation) viewed from the front and top. On the right side, the total number of π-π stacking interactions between DA4 and DA5 is shown. The different orientations of the linker and the deep insertion of DA5 led to a higher number of stacking interactions compared to those of DA4. (**C**) Water-mediated interactions observed in DA4–DNA and DA5–DNA complexes. Water-mediated inter-duplex interactions between the amino nitrogen of DA5 and the oxygen atoms of two cytosine bases from two different duplexes. The straight orientation of the DA4 linker does not allow direct water-mediated interactions with cytosine bases between the two duplexes. DA4 and DA5 are shown as orange and green sticks, respectively. Water is shown as cyan spheres, with the electron density maps for the water molecules contoured at the 1.0 σ level.

Figure 3 depicts the binding sites of DA4 and DA5 in two adjacent duplexes. The inter-duplex intercalation of diacridines at the C1pG2 and C7*pG8* sites led to extensive interactions with the terminal bases. In both crystal structures, one chromophore from DA4 and DA5 engaged in π–π stacking interactions with the C1pG2/C15pG16 steps of one duplex, whereas the other chromophore interacted with the C7*pG8*/C9*pG10* steps of the adjacent duplex (Figure 3A, B). A detailed analysis revealed that, compared to DA4, the bending of the DA5 linker resulted in more π–π stacking interactions with DNA bases. Furthermore, amino nitrogen at carbon 9 of acridine in DA5 showed a direct water-mediated interaction with cytosines C1 and C9* from two adjacent duplexes (Figure 3C). Due to the significant staggering between the two chromophores (∼60°) and the straight orientation of the linker, this direct water-mediated interaction was not observed in the DA4–DNA complex. Instead, the DA4–DNA complex exhibited a network of water molecules that interacted independently with the cytosine bases of the two duplexes. These findings suggest that DA5 may induce stronger structural perturbations in DNA than DA4, potentially leading to greater stabilizing effects.

To confirm these observations, we characterized the binding effects of the two diacridines on d(CGTATACG)_2_ DNA using circular dichroism (CD) analyses (Supplementary Figure S6A). In the absence of the compounds, the CD spectra of DNA (blue line) exhibited a negative and a positive peak at ∼250 and ∼278 nm, respectively, closely resembling the typical spectral features of B-DNA. Upon titration with a range of concentrations of both compounds, the spectra showed a decrease in CD intensity at ∼250 nm. In contrast, the intensity of the positive peak increased and shifted significantly from 278 nm to 271 nm in a concentration-dependent manner, indicating conformational changes in DNA upon ligand binding. DA5 exhibited stronger spectral changes than DA4, suggesting that DA5 has a more pronounced effect on DNA. We confirmed the stabilizing effects of the diacridines on DNA by measuring the melting temperature in the presence and absence of the compounds at a 1:4 DNA:compound ratio (Supplementary Figure S6B). Consistent with the results of CD analyses, DA5 demonstrated strong stabilizing effects, with the T_m_ values increasing by approximately 8.6°C for DA5 and 5.8°C for DA4.

### Comparison of DNA duplex conformations in the DA4–DNA and DA5–DNA complexes

**Figure 4.**
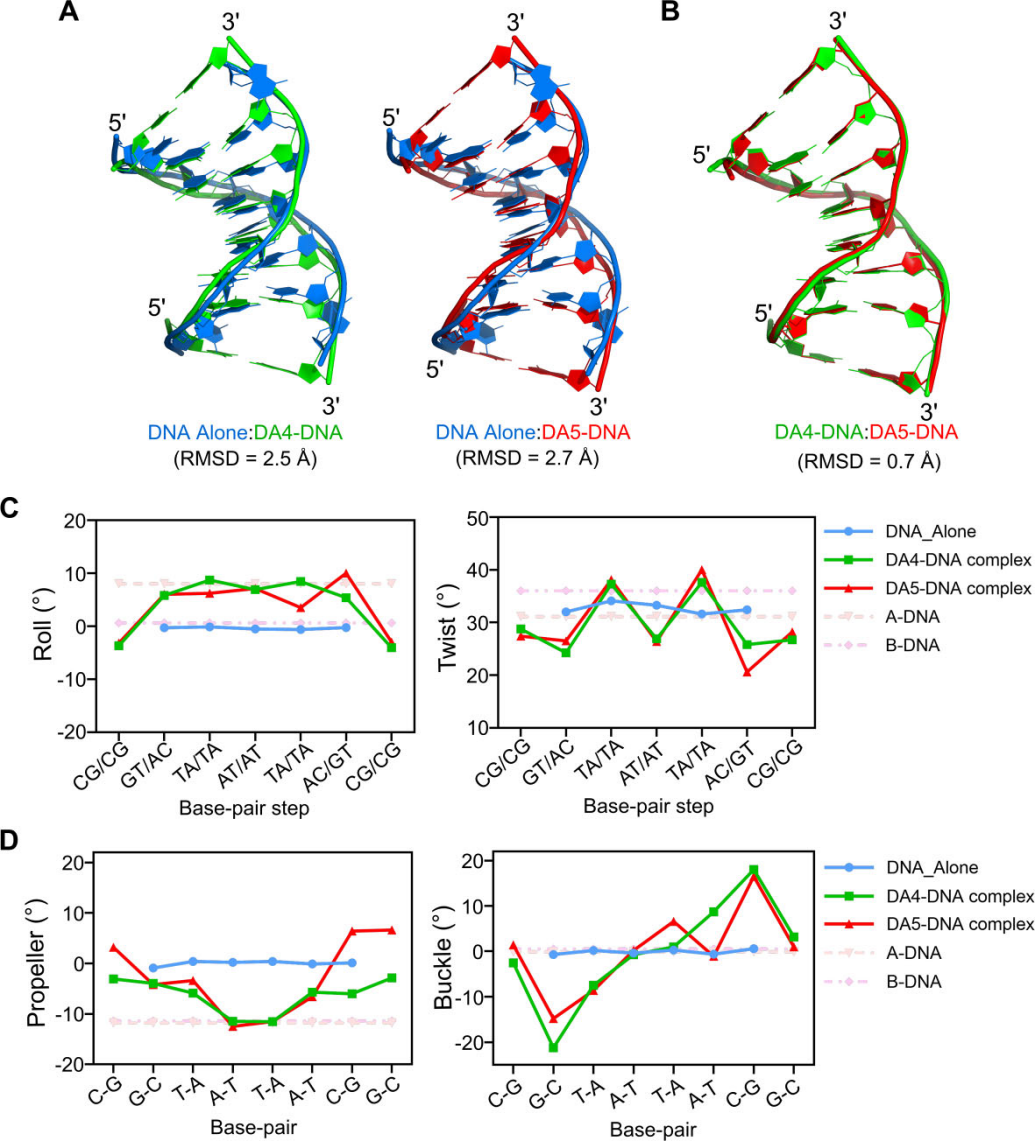
Comparison of DNA duplexes in the DA4–DNA and DA5–DNA complexes with d(CGTATACG)_2_ DNA alone. Superimposition of the crystal structures of DNA alone with the (**A**) DA4–DNA complex and DA5–DNA complexes, showing the overlap of these structures. The root-mean-square deviation (RMSD) values for 328 DNA atoms in these structures are 2.5 Å and 2.7 Å, respectively, indicating structural perturbations upon ligand binding. (**B**) Superimposition of the DNA structures of DA4–DNA and DA5–DNA complexes shows an RMSD of 0.7 Å, indicating local differences in these structures despite global similarities. Variations in the (**C**) DNA base-pair step parameters roll and twist angles and the (**D**) DNA base-pair parameters propeller and buckle angles observed in these duplexes. The values of the DNA-alone structure and standard A- and B-DNA are given for reference.

To gain detailed insight into the impact of DA4 and DA5 on the overall structural changes in DNA, we comprehensively analyzed their DNA parameters and compared them with the native DNA structure and standard A- and B-DNA values. Superimposing DA4–DNA and DA5–DNA structures onto the native DNA structure yielded root-mean-square deviation (RMSD) values of approximately 2.5 Å and 2.7 Å for 328 DNA atoms, respectively (Figure 4A). The RMSD values between DA4–DNA and DA5–DNA complexes were 0.7 Å for the same number of atoms (Figure 4B), suggesting that whereas these structures adopt right-handed conformations, they exhibit significant differences upon ligand binding. The average helical twist (h-twist) for the DNA-alone structure was approximately 33.1° ± 1.2°, whereas the h-twist values for the DA4–DNA and DA5–DNA duplexes were approximately 31.2° ± 5.8°. The average helical rise (h-rise) values for the DA4–DNA and DA5–DNA structures were approximately 1.0 Å higher than the DNA alone, indicating an increase in DNA length because of ligand intercalation. Interestingly, the roll angles of the native DNA structure were close to the standard B-DNA values (0°). Upon DA4 and DA5 binding, both complexes exhibited increased roll angles close to A-DNA values (Figure 4C). The sum of roll angles of central five steps was 35.3° and 32.8° for DA4–DNA and DA5–DNA complex structures, respectively, suggesting a sharp curvature in the backbone upon compound intercalation. Furthermore, the twist angle values of two central TA/TA steps were significantly higher (37.3° and 37.6° in DA4–DNA and 38.1° and 40° in DA5–DNA complex) than those of their adjacent base-pair steps. These alternating twist angles resulted in the overwinding of the backbone after intercalation. Thus, DA4 and DA5 binding induce substantial topological changes in DNA.

The structural differences in these complexes were even more pronounced in the terminal DNA base pairs. In the DNA-alone structure, the terminal base pairs C1–G16 and G8–C9 exhibited an unexpected distortion, failing to form a standard base pair. Instead, the cytosine bases (C1 and C9) protruded away from the helical axis, whereas the complementary guanines (G8 and G16) adopted a highly distorted extrahelical conformation with their Hoogsteen edges aligned outside the helical axis (Supplementary Figure S7). These structural differences are reflected in the altered backbone torsion angles. For example, α values changed from 123.7° to –81.2° at the C7 to G8 and C15 to G16 steps, whereas β values changed from –131.5° to –170.2° in the DNA-alone structure. The ϵ, ζ and χ angles for the terminal C1 (or C9) bases were –78.1°, 111.8° and –59°, respectively, which significantly differed from the standard values of A- and B-DNA. These differences changed the orientation of the backbone at the terminal steps and led to the fraying of the C–G base pairs. Upon binding of DA4 and DA5 to the DNA, the variations in the β-torsion angle at the terminal steps were reduced by approximately 17° and 11°, respectively, whereas the sugar pucker for the terminal G8 base changed from a C2'-*exo* to a C4'-*exo* conformation. Moreover, the glycosyl torsion angles χ for the terminal C1 (and C9) bases were –169.1° and 170.7° in the DA4–DNA and DA5–DNA complexes, respectively, which were closer to the value of A-DNA (χ, –160°) and significantly different from the value in the DNA-alone structure (χ, –59°). In addition, the sugar puckers for the C1pG2 or C9pG10 bases in the DA4–DNA complex were in C3′-*exo* and C2′-*endo* conformations, whereas in the DA5–DNA complex, they were in C2′-*endo* and C3′-*exo* conformations. Furthermore, the DNA base-pair parameter ‘propeller’ exhibited negative values of approximately –6° and –2.8° for the terminal base pairs C7–G10 and G8–C9, respectively, in the DA4–DNA complex, compared to positive values of 6.4° and 6.6° for the corresponding base pairs in the DA5–DNA complex. The terminal base pairs demonstrated significant buckling and opening in both complexes (Figure 4D). Collectively, these differences at the chromophore binding site in the two complex structures suggest that the distinct binding modes of DA4 and DA5 may lead to their specific biological effects.

### DA4 and DA5 inhibit topoisomerase II (TOP2) activity in a concentration-dependent manner

**Figure 5.**
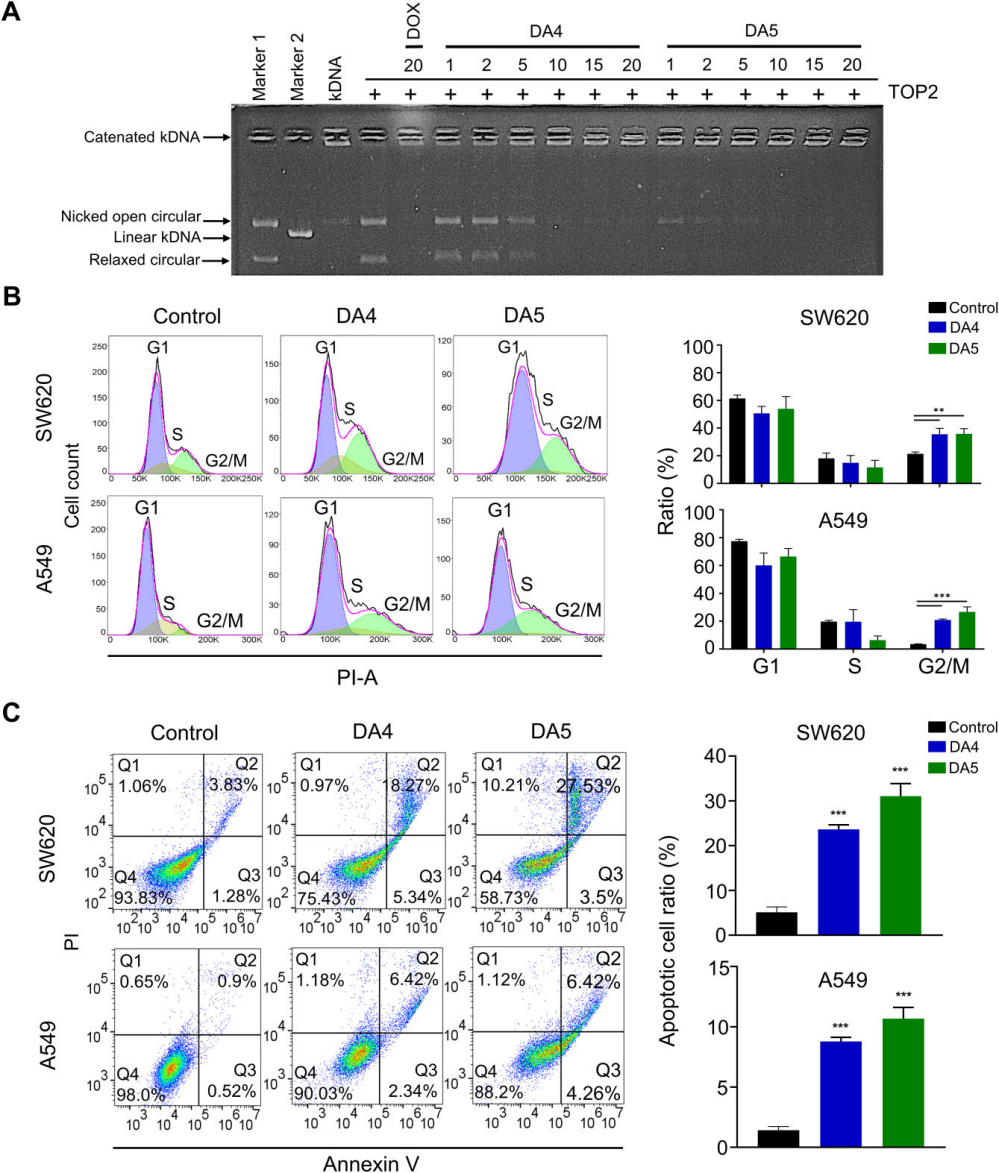
Evaluation of the anticancer activity of DA4 and DA5. (**A**) Representative gel for topoisomerase 2 (TOP2)-mediated relaxation assay. Lane 1, decatenated kDNA DNA (marker 1); lane 2, linear kDNA (marker 2); lane 3, kDNA alone; lane 4, kDNA and TOP2; lane 5, kDNA, TOP2, and 20 μM doxorubicin (DOX); lanes 6–11, kDNA, TOP2, and DA4 at 1, 2, 5, 10, 15 and 20 μM; lanes 12–17, kDNA, TOP2 and DA5 at 1, 2, 5, 10, 15 and 20 μM. (**B**) Cell cycle analysis after 12 h of treatment with 25 μM DA4 and DA5, followed by propidium iodide (PI) staining and BD Accuri™ C5 software analysis. Percentage of cells in different phases of the cell cycle. (**C**) Apoptotic SW620 and A549 cancer cells treated with 25 μM of each compound for 24 h were measured by flow cytometry with Annexin V and PI staining (*P < 0.05, **P < 0.01, ***P < 0.001).

Many of the acridine intercalators are able to inhibit TOP2 enzymes (46,47). To test whether DA4 and DA5 can inhibit the functions of topoisomerases, we examined their effects on TOP2 activities by measuring the extent of DNA relaxation mediated by this enzyme after treatment with the two diacridines (Figure 5A). Both DA4 and DA5 exhibited concentration-dependent inhibition of topoisomerase activities. DA5 can effectively reduce the amount of decatenated substrate DNA at concentrations as low as 2 μM, whereas a concentration of approximately 5 μM is required to completely inhibit TOP2 activity. This concentration is more effective than the 10 and 20 μM required by DA4 and a known TOP2 inhibitor, doxorubicin (DOX), respectively, under similar experimental conditions. On the other hand, the monointercalating precursor 9-aminoacridine (9-AA) of DA4 and DA5 showed no inhibition of TOP2 (Supplementary Figure S8A). While diacridines with longer linkers are expected to exhibit higher activity, our results showed that the TOP2 inhibitory activity of DA5 was similar to that of a nine-hydrocarbon alkyl linker chain containing bis-acridine derivative (DA9). This indicates that a five-carbon hydrocarbons linker is sufficient for the activity of an inter-duplex bis-intercalator. These findings suggest that the bis-intercalating binding mode of DA5 inhibits TOP2 activity more efficiently than a mono-intercalating agent. Neither DA4 nor DA5 showed inhibitory effects on the polymerization activity of DNA polymerase 1 (Supplementary Figure S8B), indicating that the primary mechanism of the action of diacridines may follow the inhibition of TOP2 activity.

### Anticancer evaluation of DA4 and DA5 in cancer cells and *in vivo* xenograft mouse models

**Figure 6.**
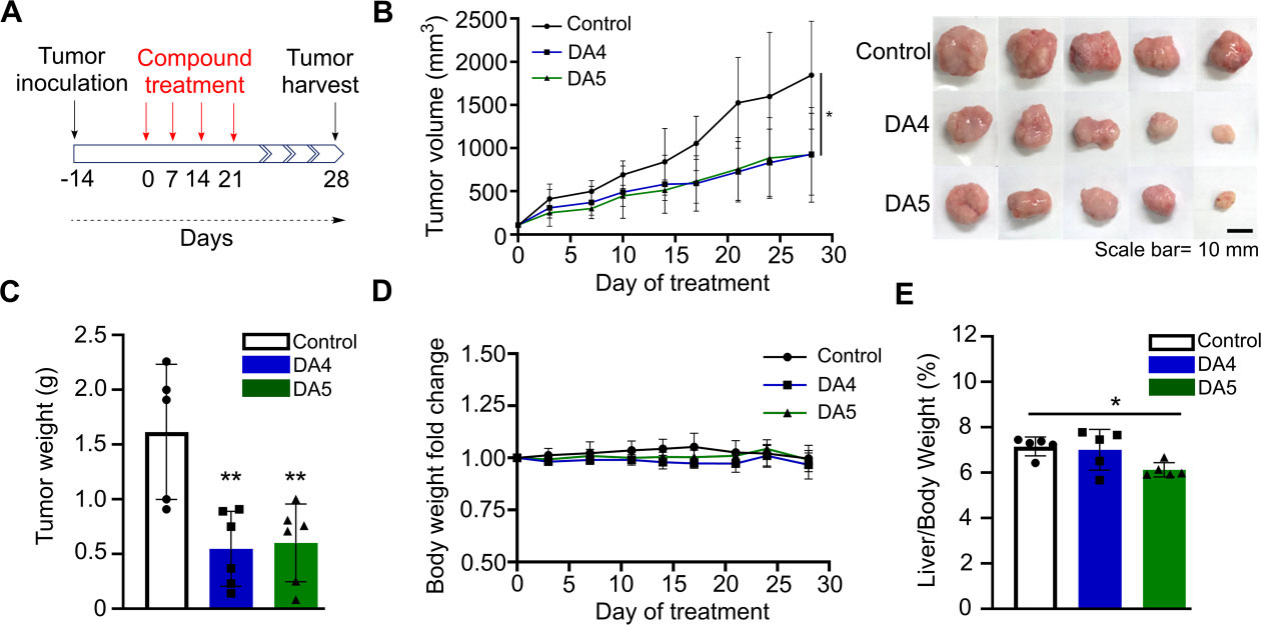
Antitumor efficacy of DA4 and DA5 in the SW620 xenograft model. (**A**) Experimental design. Seventeen subcutaneous tumor model mice with SW620 xenograft were divided into the control group (*n* = 5) and the treatment group (*n* = 6 per group). The mice in the control group received 5 mg/ml bovine serum albumin (vehicle only) and the mice in the treatment group received a dose of 1.2 mg/kg of DA4 and DA5 compounds intraperitoneally once a week for four weeks. The antitumor efficacy of DA4 and DA5 compounds was assessed in SW620 tumor models by measuring (**B**) tumor volume and (**C**) tumor weight. Scale bar represents 10 mm. (**D**) Plot showing changes in the body weight of mice from the start of treatment. (**E**) The liver/body weight ratio of mice in the treatment groups was determined at the endpoint. The error bars represent the mean ± S.D (control group: *n* = 5; DA compound treatment groups: *n* = 6 per group). Statistical significance of differences between the control and treatment groups was determined using a two-tailed test (*P > 0.05, **P > 0.01).

To understand the anticancer potential of two diacridines, we assessed the cytotoxicity of DA4 and DA5 in SW620 (colon cancer) and A549 (lung cancer) cell lines to determine their potential to inhibit cancer cell proliferation. The IC_50_ values of DA4 and DA5 in these cell lines after a 48-h treatment period ranged from 0.45 ± 0.1 to 2.9 ± 0.6 μM (Supplementary Figure S9), with DA5 exhibiting higher sensitivity in both cell lines in a dose-dependent manner. To elucidate the anticancer mechanisms of DA4 and DA5, we explored the relationship between cell proliferation inhibition and cell cycle arrest. Flow cytometry analysis of the cell cycle phase distribution after 12 h of drug treatment revealed a decrease in the number of cells in the S phase and an increase in the number of cells in the G2/M phase (Figure 5B), suggesting that both diacridines can induce G2/M phase accumulation, thus delaying cancer cell growth. Compared to DA4, the longer linker containing DA5 reduces a greater number of cells in S phase, suggesting that DA5 has stronger effects on cellular functions. The longer linker may allow DA5 to interact with DNA in a more stable or extensive manner and might resulted in greater inhibition of cellular processes. Furthermore, drug-induced cell growth inhibition may lead to apoptosis (48,49). Therefore, we performed experiments to evaluate cell death by staining cells with Annexin V-FITC and propidium iodide (PI). These experiments demonstrated that 24-h treatment with DA4 and DA5 significantly increased the number of apoptotic cells compared with the control group (Figure 5C), indicating that these compounds promote cell cycle arrest and induce apoptosis. These findings follow our earlier results, which suggested that a bis-intercalator with a more flexible linker exerts stronger anticancer effects. Moreover, we evaluated the antitumor efficacy of DA4 and DA5 *in vivo* using xenograft models of colorectal cancer with SW620 cells. The experimental schematic is shown in Figure 6A. Both diacridines significantly reduced tumor growth rate and tumor weight (Figure 6B, C), with average tumor volume reductions of 49.6% and 50% for DA4 and DA5, respectively, and tumor weight reductions of 66.1% and 62.7%, respectively. No changes in mice body weight were observed after treatment (Figure 6D), and no mice died from either DA4 or DA5 treatment. However, at the end of the experiment, a slight decrease in liver weight was observed in the DA5 treatment group (Figure 6E), possibly indicating hepatocyte toxicity, which aligns with the higher cytotoxicity observed in cell-based assays. These results suggest that the bis-intercalating diacridine compounds has potential antitumor properties.

## Discussion

Biological processes such as recombination or replication can form juxtaposed helix–helix structures and duplex crossovers (50–52). Within these crossover structures, the base pairs of the duplexes can interact with each other, resulting in junctions (53). Previous studies have shown that the diacridine intercalators with short and flexible linkers have a potential to bind to DNA–DNA junctions and cross-linking into DNA duplex–duplex contacts, inhibiting topoisomerases and exerting anticancer effects (28,42,43,54,55). However, the structural basis for targeting DNA duplex–duplex junction sites by bis-intercalators has not been clearly understood.

This study used d(CGTATACG)_2_ DNA, which displays unique structural characteristics at the terminal CG sites by forming intertwined helix–helix junction conformation. The terminal CG base pairs in this structure are involved in the formation of extrahelical tetraplex base–base arrangements. A similar structure containing four bases at the helix–helix junction has been previously reported (56). The analysis of these structures indicates that the terminal tetraplex base pairing enables DNA to form a continuous end-to-end helix–helix junction, which represents a partial structure of a crossover site in catenated DNA. The proximity of duplex–duplex contacts at CG base pairs makes it an ideal target for the binding of CG-specific bidirectional bis-intercalating ligands. We investigated the structural basis of two diacridine derivatives formed by linking two 9-aminoacridine chromophores with four- and five-carbon hydrocarbon linkers. These derivatives exhibited bis-intercalation between two adjacent DNA duplexes, inducing severe topological changes in DNA. Therefore, a side-by-side duplex arrangement is formed, in which each end of the double helix is stabilized by the diacridine compounds. Cross-linking of DNA duplexes results in a transition from the B-form to an A-form-like conformation, accompanied by bending and overwinding in the backbone. These effects significantly differ from those observed in the previously reported DNA cross-linking complex structure of the bidirectional bis-intercalator, bis(9-aminoacridine-4-carboxamide) (42). In that study, bis-intercalation into two adjacent duplexes produced a typical roll angle of approximately 10° in DNA. In contrast, the duplexes in the current DA4–DNA and DA5–DNA complexes showed a significant curvature in the backbone (sum of roll angles at the central region is 35.3° and 32.8° for DA4–DNA and DA5–DNA complexes, respectively). This indicates that the DNA backbone bends significantly upon the intercalation of these compounds. These structural variations led to distinct water-mediated interactions between DA4 and DA5 with DNA bases. In DA4–DNA complex, three water molecules mediate indirect interactions between two inter-strand cytosine bases and the DA4. In contrast, the DA5–DNA complex has a single water molecule directly bridging two inter-duplex cytosines and DA5. Previous studies have shown that water significantly enhances the binding affinity of DNA-binding ligands (57,58). In the DA4–DNA complex structure, the straight linker and highly tilted chromophore of DA4 create space for accommodating water. However, the increased distance between the amino group of the linker and the keto group of cytosine leads to indirect and weaker interactions. The bent linker and less tilted chromophore of DA5 bring the amino atoms closer to cytosines, favoring a direct water-mediated interaction which likely results in a more stable complex with stronger binding interactions. The biophysical data support these observations. At the cellular level, the biological effects of DA5 are expected to be more pronounced than those of DA4. Thus, the structural changes induced by DA4 or DA5 result from ligand binding, highlighting their potential biological importance.

Furthermore, we examined the interactions of DA4 and DA5 with various DNA sequences, including hairpin and bulge-containing duplexes, triplex- and quadruplex-forming sequences, using CD spectral analysis (Supplementary Figure S10). Both DA4 and DA5 showed minimal alterations in CD spectra characteristics of triplex and hybrid topology-forming human telomeric G-quadruplex sequences at different ligand concentrations, suggesting that the two diacridines do not affect these structures (59,60). In contrast, the diacridines show strong spectral changes in the negative and positive peak intensities for the hairpin- and bulge-containing duplexes, similar to d(CGTATACG)_2_ DNA. These changes in the intensities of the CD spectra can be attributed to the stable complex formation of DA4 and DA5 with these duplexes (61,62). These results suggest that the bis-intercalating diacridines prefer to intercalate into duplex conformations, including hairpin or bulge structures, which can easily form different junction or cruciform structures. Since, TOP2 play an important role in recognizing the specific geometries of DNA, including hairpin and cruciform structures (9), the possible biological activity of these compounds may be due to their TOP2 inhibitory activity. However, further studies will require to confirm the mechanisms of topoisomerase inhibition and DA4/DA5 binding. Our *in vitro* results showed that the current diacridines can induce G2/M phase accumulation and trigger apoptosis to inhibit cancer cell growth. The results also indicate that DA5 has more pronounced anticancer effects than DA4, likely owing to its enhanced stability and strong DNA binding interactions. Overall, our results demonstrate the potential biological consequences of the current diacridines and their structural basis for cross-linking DNA duplex–duplex contact structures.

In conclusion, in this study we have shown that a unique DNA–DNA contact structure is formed at the interface of two duplexes that resembles the characteristics of catenated DNA. This structure provides an appropriate target for bidirectional bis-intercalator binding with a tetraplex base-pair junction. Notably, bis-intercalation of diacridine causes substantial changes in DNA topology. DNA–DNA contacts are present in all cells, making them a potential target for anticancer drug discovery. This study provides structural insights that can guide the development of more effective derivatives and pave the way for targeting DNA–DNA duplex contacts through bis-intercalation.

## Supplementary Material

gkae643_Supplemental_File

## Data Availability

The atomic coordinates and structure factors for the reported crystal structures have been deposited in the Protein Data Bank under the accession numbers 8W76, 8WQ7 and 8W7W for DNA alone, DA4–DNA and DA5–DNA complex structures, respectively.
