# Supplementary material for: Targeting DNA junction sites by bis-intercalators induces topological changes with potent antitumor effects
Source: Nucleic Acids Res. 2024 Jul 22;52(15):9303–16. doi: 10.1093/nar/gkae643 (PMC11347135; doi:10.1093/nar/gkae643)
Supplement: gkae643_Supplemental_File [file gkae643_supplemental_file.pdf]

Supplementary Information

Targeting DNA junction sites by bis-intercalators induces topological changes with potent antitumor effects

Shih-Chun Huang[1,2,†], Chia-Wei Chen[3,†], Roshan Satange[2,†], Chang-Chih Hsieh[4], Chih-Chun Chang[5], Shun-Ching Wang[1,2], Chi-Li Peng[2], Tai-Lin Chen[6], Ming-Hsi Chiang[4], Yih-Chern Horng[3,*] and Ming-Hon Hou[1,2,5,7,*]

* To whom correspondence should be addressed. Tel: +886 4 2284 0338 (Ext 7011); Fax: +886 4 2285 9329; Email: mhho@nchu.edu.tw and ychorng@cc.ncue.edu.tw

† Authors contributed equally

Table of Contents

A. Supplementary Note 2

B. Supplementary Figures 3

C. Supplementary Tables 10

D. Supplementary References 15

A. Supplementary Note

General procedure for compound synthesis: Commercially available chemicals, including 9-chloroacridine, 1,5-diaminopentane, and 1,4-diaminobutane, were purchased from Sigma-Aldrich or TCI and used as received. All reagents were analytical grade and were used without further purification. Ethanol (EtOH) was distilled under nitrogen, using CaH_2 as a drying agent, and stored in N_2-filled reservoirs with four molecular sieves before use. ^{1}H nuclear magnetic resonance (NMR) spectra were collected on a Bruker Avance 300 spectrometer. Chemical shifts for ^{1}H spectra were recorded in ppm relative to the residual proton (1H dimethyl sulfoxide [DMSO]-d_6: δ 2.50). Density functional theory calculations were performed using the Gaussian 09 program. Geometry optimizations were performed with the B3LYP functional and 6-31G* basis sets. The solvation free energy was investigated using the self-consistent reaction field and solvation model (1). Elemental analysis and mass spectrometry analysis were performed on a Heraeus CHN-OS Rapid Elemental Analyzer and a JEOL JMX-SX/SX 102A Mass Spectrometer, respectively, at the Instruments Center of National Chung Hsing University, Taiwan.

Chemical synthesis

Synthesis of DA4: 9-Chloroacridine (500 mg, 2.3 mmol) and 1,4-diaminobutane (98 mg, 1.11 mmol) were dissolved in 20 mL of dry EtOH. The solution was then refluxed at 80 °C overnight. Upon the completion of the reaction, the resulting solution was cooled to room temperature (approximately 25 °C), and 100 mL of diethyl ether was added to the solution to wash the precipitate. The precipitate was then filtered and dried under vacuum, resulting in DA4 (354 mg, 80% yield). The ^{1}H NMR findings (DMSO-d6) for DA4 were as follows: δ 8.47 (d, 2H), 7.92 (t, 2H), 7.82 (d, 2H), 7.45 (t, 2H), 4.14 (t, 2H), 2.01 (t, 2H). The calculated values for $C_{30}H_{26}N_4$ in the elemental analysis were C = 81.42, H = 5.92, and N = 12.66, whereas the actual values were C = 81.53, H = 5.98, and N = 12.60. The calculated m/z value for $C_{30}H_{27}N_4$ $[M+H]^+$ in high-resolution mass spectrometry (HRMS) was m/z = 443.2230, whereas the actual value was m/z = 443.2223.

Synthesis of DA5: 9-Chloroacridine (500 mg, 2.3 mmol) and 1,5-diaminopentane (114 mg, 1.1 mmol) were dissolved in 20 mL of dry EtOH. The solution was refluxed at 80 °C overnight. Upon the completion of the reaction, the resulting solution was cooled to room temperature, and 100 mL of diethyl ether was added to wash the precipitate. The precipitate was filtered and dried under vacuum to produce DA5 (343 mg, 75%). The ^{1}H NMR findings (DMSO-d6) for DA5 were as follows: δ 8.36 (d, 2H), 7.75 (m, 4H), 7.32 (t, 2H), 3.91 (t, 2H), 1.83 (t, 2H), 1.47 (m, 1H). The calculated values for $C_{31}H_{28}N_4$ in the elemental analysis were C = 81.55, H = 6.18, and N 12.27, whereas the actual values were C = 81.53, H = 6.47, and N = 12.50. The calculated m/z value for $C_{31}H_{29}N_4$ $[M+H]^+$ in HRMS was m/z = 457.2387, whereas the actual value was m/z = 457.2383.

B. Supplementary Figures

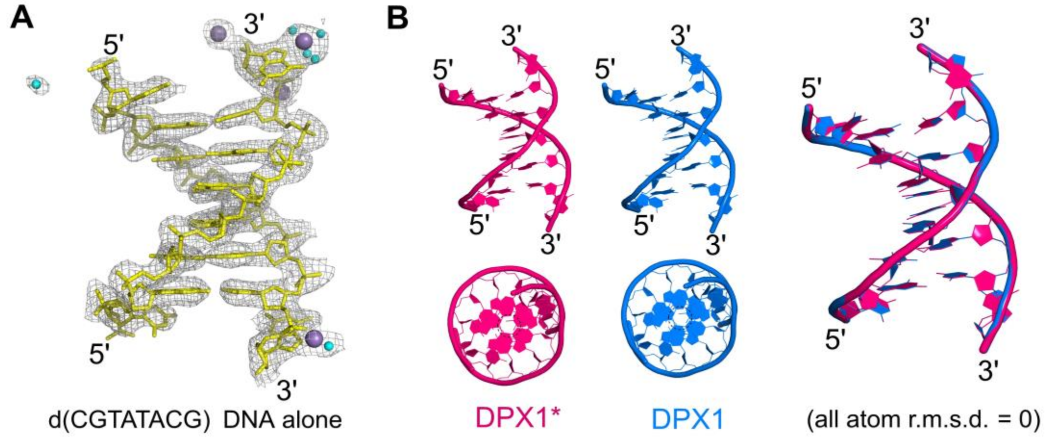

Figure S1: Crystal structure d(CGTATACG)$_2$ DNA alone. (A) 2F_o-F_c electron density maps of the refined structure of d(CGTATACG)$_2$ DNA duplex in an asymmetric unit contoured at 1.0 σ level. **(B)** Comparison of two adjacent duplexes in the crystal symmetry shows identical duplexes view from the side and top. The superposition of these duplexes shows a root mean square (R.M.S.) deviation of 0.001 Å.

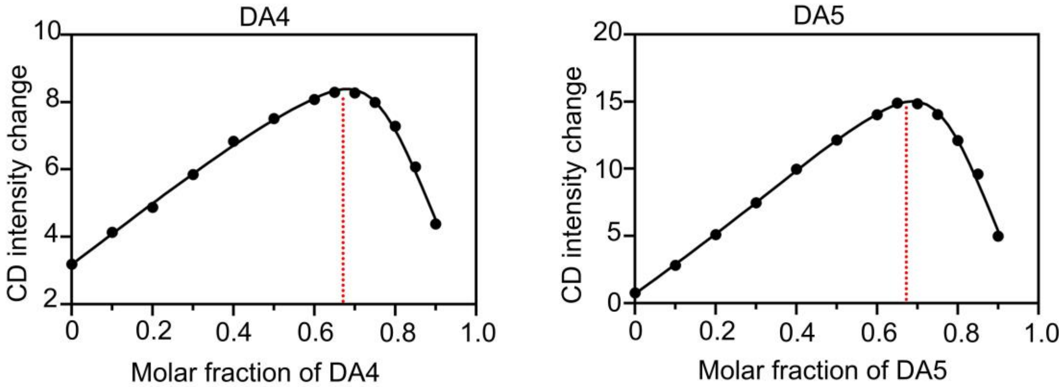

Figure S2: Analysis of binding stoichiometry in solution of DA4 and DA5 with d(CGTATACG)$_2$. Job-type titration plot for DA4 and DA5 in a buffer containing 50 mM sodium cacodylate trihydrate (pH 7.3) and 5 mM magnesium chloride hexahydrate at 25°C. The total concentration of compounds (DA4 or DA5) and DNA was set at 100 µM. The plot at 272 nm shows a distinctive maximum at about 0.66 molar fraction of compounds, indicating approximately two molecules of DA4 or DA5 bind to a duplex DNA.

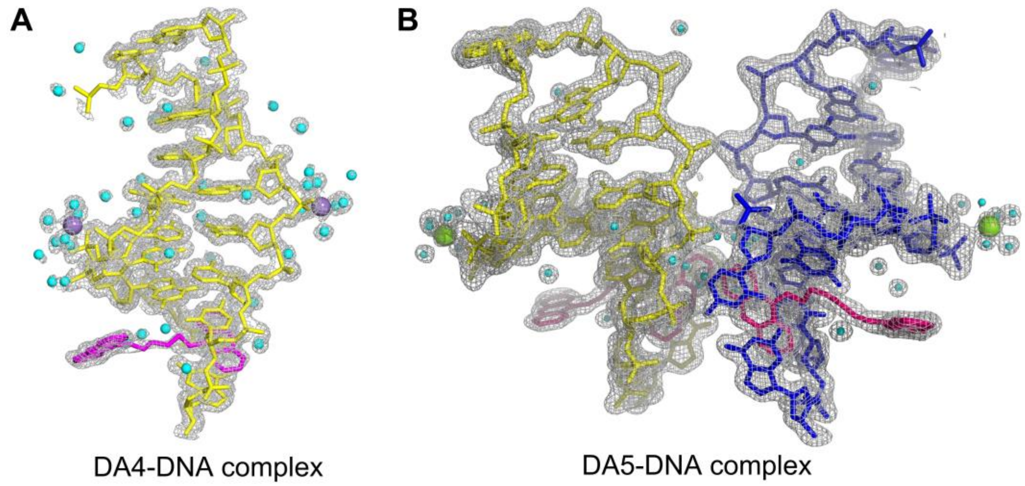

Figure S3: $2F_o$-F_c electron density maps of the refined structures of DA4-DNA and DA5-DNA complexes contoured at 1.0 σ level. The d(CGTATACG)$_2$ DNA is complexed with **(A)** DA4 (DA4-DNA complex) and **(B)** DA5 (DA5-DNA complex). DNA backbones are represented in yellow and blue sticks while compounds are shown in pink stick representation. Metal ions including manganese (purple), magnesium (green) and waters (cyan) are shown in spheres.

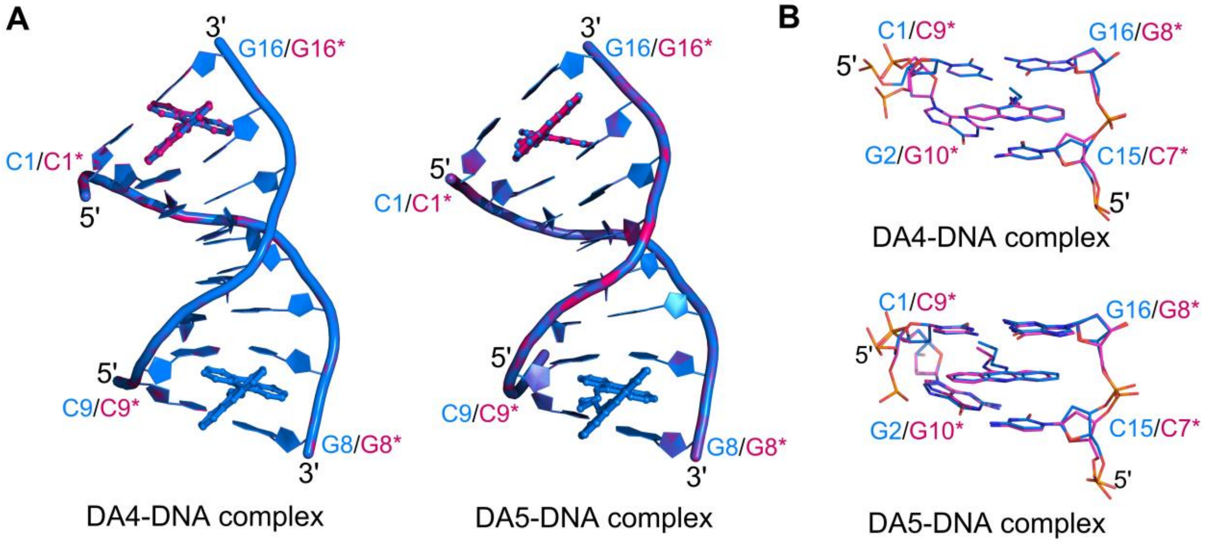

Figure S4: Superimposition of DA4-DNA and DA5-DNA complexes. (A) Overlay of two adjacent DNA duplexes (blue and pink colored cartoons) in the DA4-DNA complex (left panel) and DA5-DNA complex (right panel), showing that these duplexes are identical. **(B)** Superimposition of the upper C1-G16/G2-C15 chromophore binding site of one duplex (blue colored sticks) with the C9*-G8*/G10*-C7* site (pink colored sticks) in another duplex showing the differences in interduplex bis-intercalation of DA4 and DA5 structures.

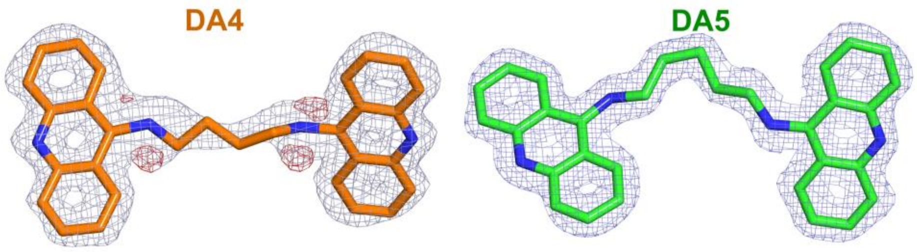

Figure S5: Correlation between the quality of the electron density map and the atomic models of DA4 and DA5 in two crystal structure complexes. Shown is the refined 2mF$_o$-DF$_c$ maximum likelihood-weighted Fourier electron density map in ccp4 format, countered at the 1.0 σ level with a 2.0 Å carve radius (light blue mesh). The mF$_o$-DF$_c$ difference map (red mesh at 3.0 σ) shows a good fit of the geometries of DA4 (orange sticks) and DA5 (green sticks) in the two crystal structures.

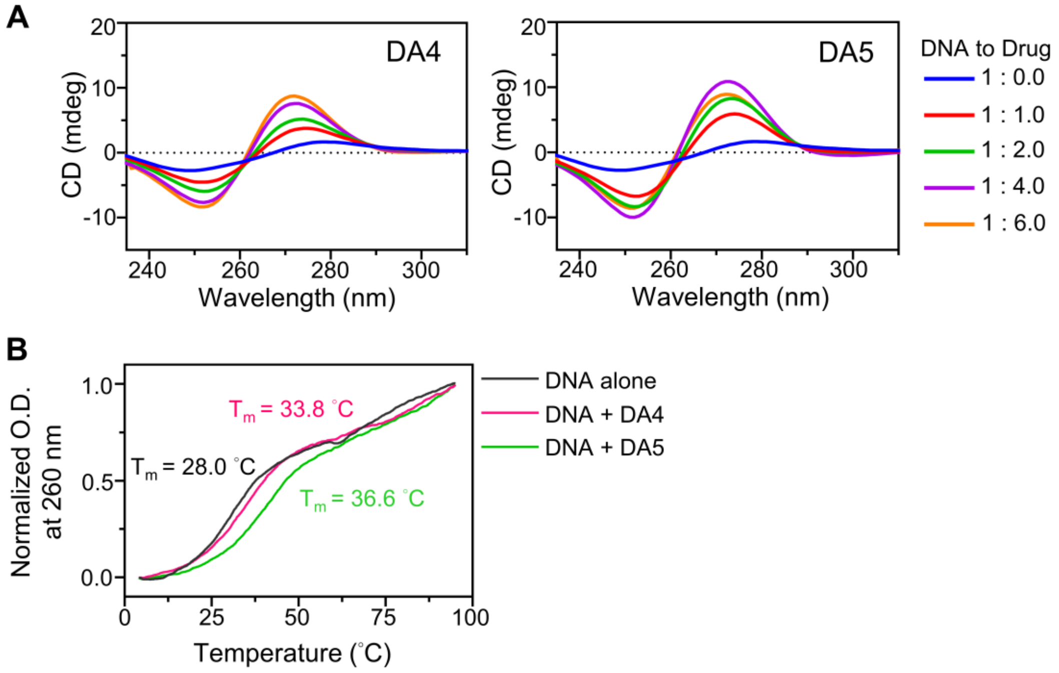

Figure S6: Stabilizing effects of DA4 and DA5 on d(CGTATACG)$_2$ DNA analyzed by circular dichroism (CD) and melting temperature (T$_m$). (A) CD spectra of d(CGTATACG) in the presence of different ratios of DA4 and DA5. Spectra were recorded in the presence of 20 µM oligonucleotides prepared in a buffer containing 20 mM sodium cacodylate (pH 7.3), 100 mM KCl and 5 mM magnesium chloride. **(B)** Effects of DA4 and DA5 on stability of d(CGTATACG)$_2$ DNA. 3 µM DNA duplex was used to determine the melting temperature (T$_m$) in absence (black line) and presence (pink and green lines) of ligands at DNA:ligand ratio of 1:4 in the same buffer as the CD experiment. This ratio was chosen as CD spectra exhibited saturation at 278 nm after the addition of four equivalents of DA5.

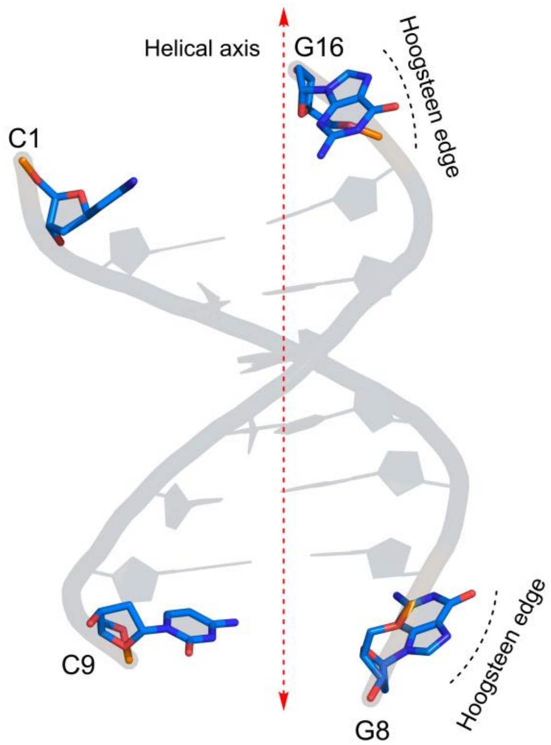

Figure S7: Terminal base pair flipping. Flipping of cytosine (C1 and C9) and guanine (G8 and G16) away from helical axis is shown in DNA alone structure of d(CGTATACG)$_2$.

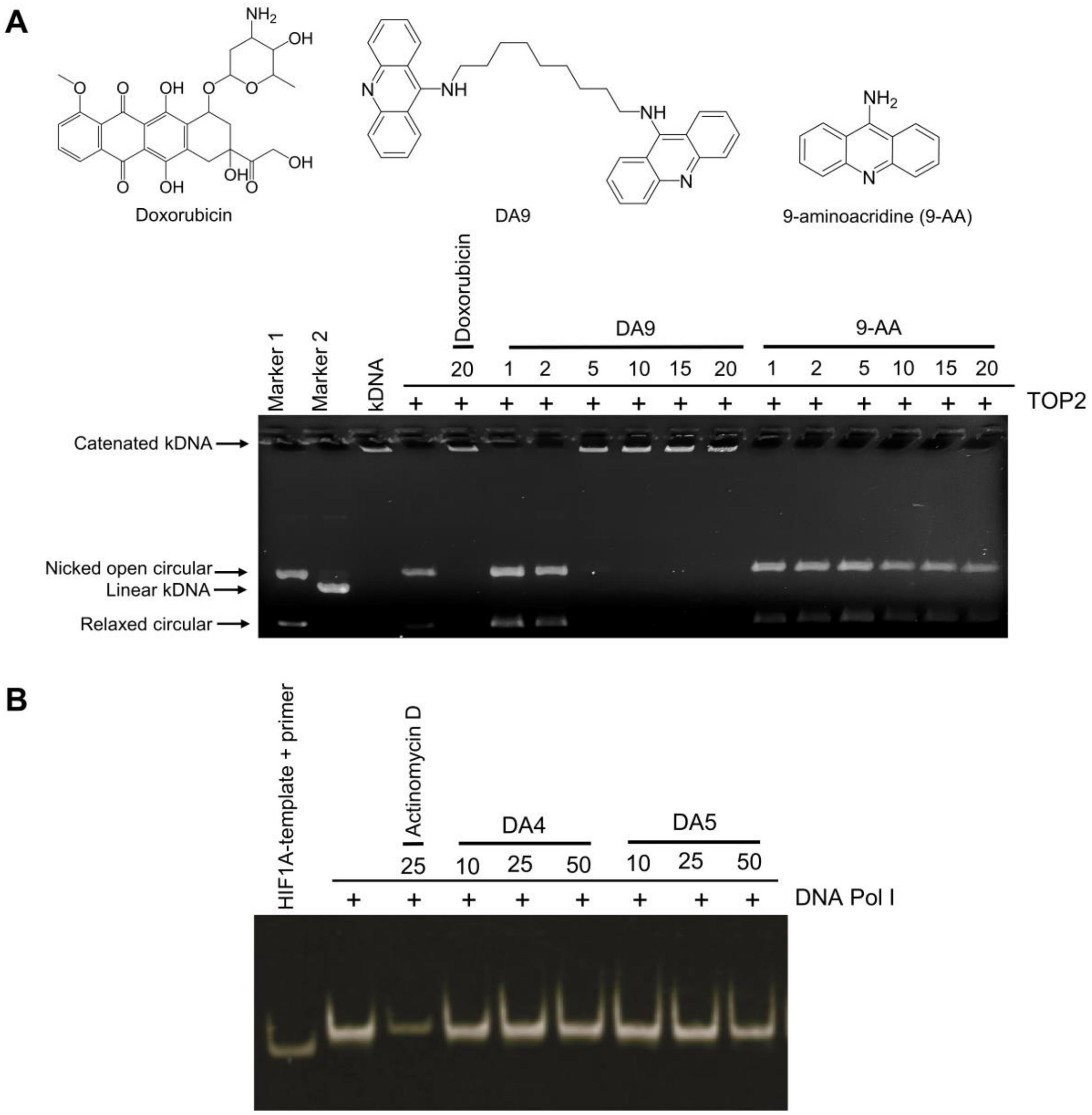

Figure S8: Human DNA topoisomerase 2 (TOP2) and polymerase I (Pol I) activity assay. (A) Top: Chemical structures of DNA mono-intercalator doxorubicin, long hydrocarbon linker chain containing bis-intercalator DA9, and a mono-intercalating precursor of diacridine compounds, 9-aminoacridine (9-AA) used in TOP2 inhibition assay. Bottom: Representative gel for TOP2-mediated relaxation assay. Lane 1, decatenated kDNA DNA (marker 1); lane 2, linear kDNA (marker 2); lane 3, kDNA alone; lane 4, kDNA and TOP2; lane 5, kDNA, TOP2, and 20 μM doxorubicin; lanes 6–11, kDNA, TOP2, and DA9 at 1, 2, 5, 10, 15, and 20 μM; lanes 12–17, kDNA, TOP2, and 9-AA at 1, 2, 5, 10, 15, and 20 μM. **(B)** Representative gel for DNA Pol I assay. A DNA template containing HIF1A promoter sequence was used. Lane 1, the control containing only HIF1A template and primer; lane 2, HIF1A template and primer with DNA polymerase I; lane 3, HIF1A template and primer with Pol I incubated with 25 μM actinomycin D; Lane 4-6, HIF1A template and primer with DA4 at 10, 25, 50 μM, incubated with Pol I; Lane 7-9, HIF1A template and primer with DA5 at 10, 25, 50 μM, incubated with Pol I.

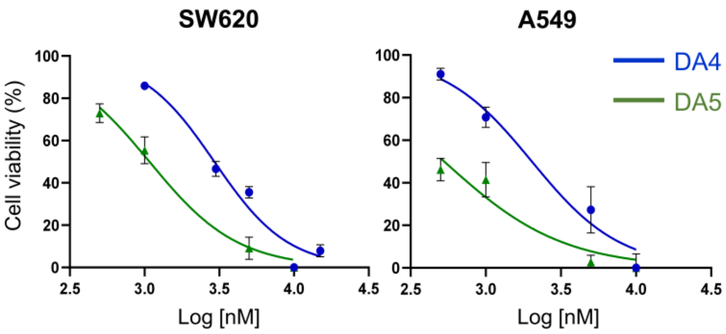

Cancer cell lines	IC$_{50}$ (µM) DA4	IC$_{50}$ (µM) DA5
SW620	2.9±0.6	1.1±0.2
A549	2.0±0.6	0.45±0.1

Figure S9: Cytotoxicity of DA4 and DA5 on SW620 and A549 cancer cells. The IC$_{50}$ values against the proliferation of cancer cells are determined for 48 h of treatment for three independent experiments. Graphs show dose–response curves after treatment of compounds.

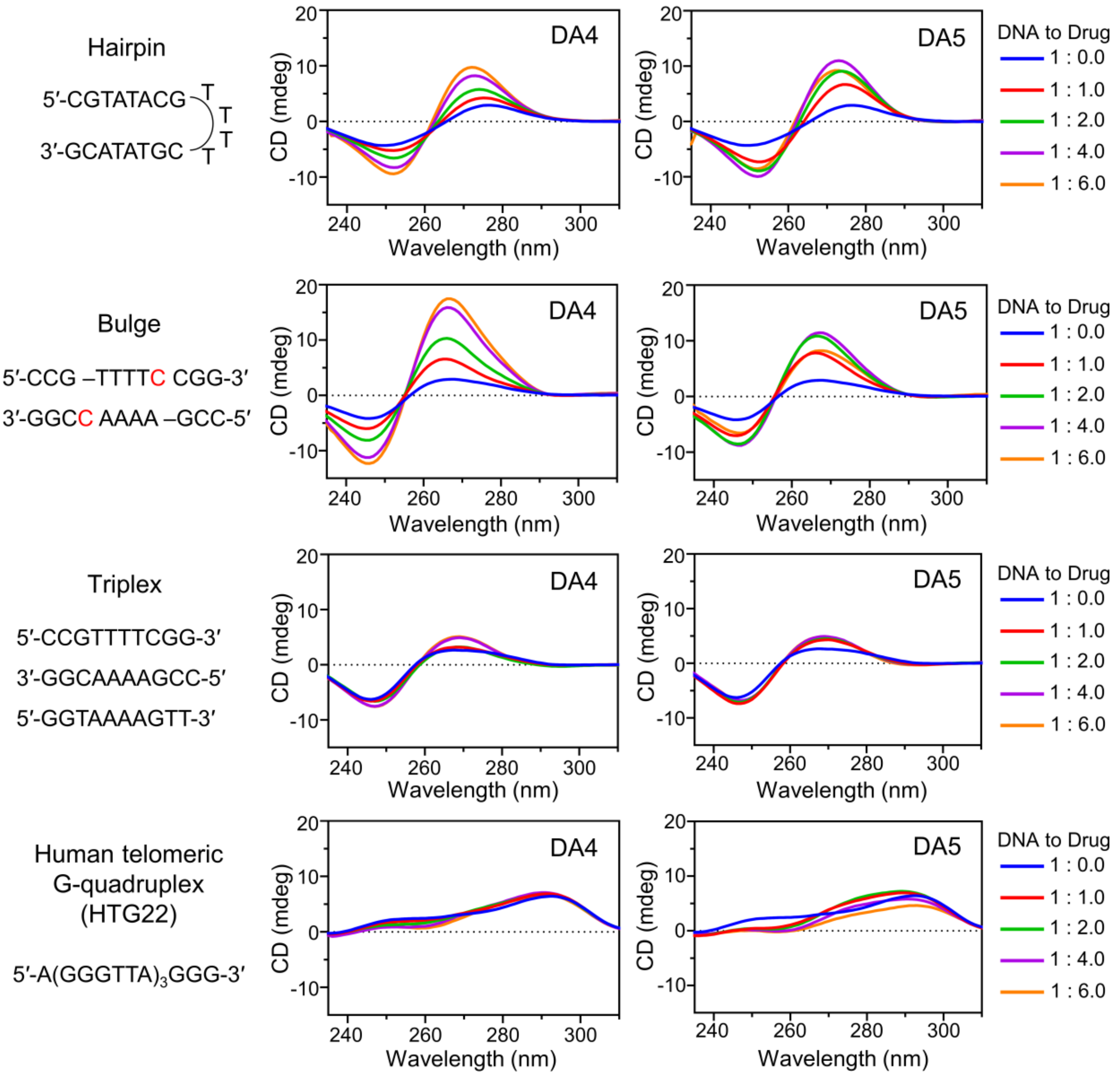

Figure S10: CD spectra analysis of different DNA oligonucleotide sequences with DA4 and DA5. CD spectra of hairpin-, bulge-, triplex- and quadruplex-containing oligonucleotides (the sequences are shown on the left in the figure) in the presence of different ratios of DA4 and DA5. The spectra show that both DA4 and DA5 can effectively interact with hairpin- and bulge-containing CG-rich sequences and significantly alter DNA conformation, similar to CGTATACG duplex DNA CD spectra. DA4 and DA5 did not show strong alterations in the spectra characteristic of triplex and human-telomeric (HTG22) hybrid G-quadruplex (K^+ form) forming sequences. Spectra were recorded in presence of 20 μM oligonucleotides prepared in a buffer containing 20 mM sodium cacodylate (pH 7.3), 100 mM KCl and 5 mM magnesium chloride.

C. Supplementary Tables

Table S1. Crystallographic and refinement statistics of the structures presented in this study.

**Data collection statistics**			
	**d(CGTATACG)$_2$ duplex**	**d(CGTATACG)$_2$ with DA4**	**d(CGTATACG)$_2$ with DA5**
Complex name	**DNA only**	**DA4-DNA complex**	**DA5-DNA complex**
Beamline	NSRRC TLS 15A, Taiwan	NSRRC TLS 15A, Taiwan	NSRRC TLS 15A, Taiwan
Detector type	MX300HE	MX300HE	MX300HE
Wavelength [Å]	1.0000	1.0000	1.00000
Data collection temperature [K]	100	100	100
Space group	$P4_12_12$	$C222_1$	$P2_1$
**Cell dimensions**			
a, *b*, *c* [Å]	37.5, 37.5, 77.9	34.4, 34.4, 69.0	24.1, 72.0, 24.1
α, *β*, *γ* [°]	90, 90, 90	90, 90, 90	90, 92.6, 90
Resolution range [Å]*	30.0-2.70 (2.70-2.80)	30.0-1.58 (1.58-1.64)	30.00-1.58 (1.64-1.58)
Total reflections	16717	39709	40205
Unique reflections	1753	5894	11068
Completeness [%]*	99.3 (100)	99.5 (100.0)	96.4 (91.0)
Mean I/σ [I]*	33.9 (20.9)	44.0 (19.1)	45.7 (7.6)
R-merge [%]*	0.033 (0.12)	0.034 (0.10)	0.17 (0.02)
Redundancy*	9.5 (10.5)	6.7 (7.0)	3.6
**Refinement statistics**			
R-work/*R*-free	0.22/0.26	0.21/0.24	0.21/0.22
No. of non-solvent atoms	332	364	728
No. of solvent atoms	6	42	39
Average B-factor [$Å^2$]	52.5	21.6	28.9
R.m.s.d bond lengths [Å]	0.004	0.008	0.01
R.m.s.d bond angles [°]	0.64	1.2	1.1
PDB code	8W76	8WQ7	8W7W

*Outer shell statistics are shown in parentheses.

Table S2: DNA torsion angles and sugar pucker of **(A)** d(CGTATACG)$_2$ duplex (DNA only), **(B)** d(CGTATACG)$_2$ with DA4 (DA4-DNA), **(C)** d(CGTATACG)$_2$ with DA5 (DA5-DNA) complexes analyzed by Curves+ online web server. Values of standard A-DNA and B-DNA are given for comparison.

**(A)**	**DNA only**							
	**α**	**β**	**γ**	**δ**	**ε**	**ζ**	**χ**	**pucker**
A-DNA	-65	180	60	81	-148	-71	-160	C3'*en*
B-DNA	-41	-136	40	139	-133	-157	-100	C2'*en*
**Base number**								
**C1**	----	171.9	67.4	155.2	-78.1	111.8	-59.0	C2'*en*
**G2**	34.7	143.0	-82.5	167.0	-179.2	-90.3	-102.1	C3'*ex*
**T3**	-88.3	161.8	81.4	111.5	-155.7	-118.8	-139.1	C1'*ex*
**A4**	-8.3	161.5	0.4	142.3	-175.3	-106.0	-96.3	C2'*en*
**T5**	-72.5	160.0	76.4	102.1	-160.0	-93.0	-133.2	O4'*en*
**A6**	-52.7	163.6	42.6	110.0	-158.8	-124.9	-132.6	C1'*ex*
**C7**	123.7	-131.5	-168.7	106.9	-142.9	-42.7	-155.0	O4'*en*
**G8**	-81.2	-170.2	52.2	101.5	----	----	-82.0	C2'*ex*
C9	----	171.9	67.4	155.2	-78.1	111.8	-59.0	C2'*en*
G10	34.7	143.0	-82.5	167.0	-179.2	-90.3	-102.1	C3'*ex*
T11	-88.3	161.8	81.4	111.5	-155.7	-118.8	-139.1	C1'*ex*
A12	-8.3	161.5	0.4	142.3	-175.3	-106.0	-96.3	C2'*en*
T13	-72.5	160.0	76.4	102.1	-160.0	-93.0	-133.2	O4'*en*
A14	-52.7	163.6	42.6	110.0	-158.8	-124.9	-132.6	C1'*ex*
C15	123.7	-131.5	-168.7	106.9	-142.9	-42.7	-155.0	O4'*en*
G16	-81.2	-170.2	52.2	101.5	----	----	-82.0	C2'*ex*

**(B)**	**DA4-DNA complex**							
	**α**	**β**	**γ**	**δ**	**ε**	**ζ**	**χ**	**pucker**
A-DNA	-65	180	60	81	-148	-71	-160	C3'*en*
B-DNA	-41	-136	40	139	-133	-157	-100	C2'*en*
**Base number**								
**C1**	----	-16.4	-111.6	145.6	-101.4	-97.7	-169.1	C3'*ex*
**G2**	-50.6	174.5	35.0	143.7	-178.8	-95.0	-91.4	C2'*en*
**T3**	-57.8	166.7	49.8	102.7	-174.1	-112.3	-121.2	O4'*en*
**A4**	-50.6	171.8	50.3	146.5	-164.0	-96.0	-101.7	C2'*en*
**T5**	-60.9	164.5	46.1	97.3	-176.8	-88.8	-120.5	O4'*en*
**A6**	-63.2	-175.3	48.5	141.9	-145.3	-136.2	-86.3	C2'*en*
**C7**	86.0	-136.1	-155.6	97.2	-138.6	-76.1	-133.5	C4'*ex*
**G8**	-75.3	-153.0	59.7	90.9	----	----	-81.1	C4'*ex*
C9	----	-16.4	-111.6	145.6	-101.4	-97.7	-169.1	C3'*ex*
G10	-50.6	174.5	35.0	143.7	-178.8	-95.0	-91.4	C2'*en*
T11	-57.8	166.7	49.8	102.7	-174.1	-112.3	-121.2	O4'*en*
A12	-50.6	171.8	50.3	146.5	-164.0	-96.0	-101.7	C2'*en*

T13	-60.9	164.5	46.1	97.3	-176.8	-88.8	-120.5	O4'*en*
A14	-63.2	-175.3	48.5	141.9	-145.3	-136.2	-86.3	C2'*en*
C15	86.0	-136.1	-155.6	97.2	-138.6	-76.1	-133.5	C4'*ex*
G16	-75.3	-153.0	59.7	90.9	----	----	-81.1	C4'*ex*

**(C)**	**DA5-DNA complex**							
	**α**	**β**	**γ**	**δ**	**ε**	**ζ**	**χ**	**pucker**
A-DNA	-65	180	60	81	-148	-71	-160	C3'*en*
B-DNA	-41	-136	40	139	-133	-157	-100	C2'*en*
**Base number**								
**C1**	----	110.2	146.4	158.5	-101.7	-88.6	-170.7	C2'*en*
**G2**	-67.2	-179.9	43.3	144.8	178.3	-99.6	-88.6	C3'*ex*
**T3**	-53.4	168.9	48.2	116.3	-170.2	-125.4	-114.4	O4'*en*
**A4**	-48.0	166.8	49.6	145.5	-166.9	-91.2	-99.4	C1'*ex*
**T5**	-60.8	167.0	38.1	100.8	177.9	-99.7	-110.9	C2'*en*
**A6**	-52.5	164.2	46.6	95.2	-151.6	-81.0	-127.9	C1'*ex*
**C7**	-57.5	163.0	53.5	107.5	-167.7	-99.0	-113.2	C3'*en*
**G8**	129.6	152.2	-128.0	109.5	----	----	-89.3	C4'*ex*
C9	----	110.2	146.4	158.5	-101.7	-88.6	-170.7	C2'*en*
G10	-67.2	-179.9	43.3	144.8	178.3	-99.6	-88.6	C3'*ex*
T11	-53.4	168.9	48.2	116.3	-170.2	-125.4	-114.4	O4'*en*
A12	-48.0	166.8	49.6	145.5	-166.9	-91.2	-99.4	C1'*ex*
T13	-60.8	167.0	38.1	100.8	177.9	-99.7	-110.9	C2'*en*
A14	-52.5	164.2	46.6	95.2	-151.6	-81.0	-127.9	C1'*ex*
C15	-57.5	163.0	53.5	107.5	-167.7	-99.0	-113.2	C3'*en*
G16	129.6	152.2	-128.0	109.5	----	----	-89.3	C4'*ex*

Table S3: DNA base pair and base pair step parameters of **(A)** d(CGTATACG)$_2$ duplex (DNA only), **(B)** d(CGTATACG)$_2$ with DA4 (DA4-DNA), **(C)** d(CGTTAACG)$_2$ with DA5 (DA5-DNA) complexes analyzed by Web3DNA online web server.

**(A)**	**DNA only**						
	Base pair parameter						
		**Buckle (°)**	**Propeller (°)**	**Opening (°)**	**Shear (Å)**	**Stretch (Å)**	**Stagger (Å)**
**Base-pair**	**Base-pair identity**						
**C1-G16**	1	-	-	-	-	-	-
**G2-C15**	2	-0.7	-0.9	0	-0.7	-0.1	0
T3-A14	3	0.2	0.4	0.4	0.2	0.4	0.4
A4-T13	4	-0.4	0.2	-0.4	-0.4	0.2	-0.4
T5-A12	5	0.3	0.4	-0.2	0.3	0.4	-0.2
A6-T11	6	-0.6	-0.1	0.1	-0.6	-0.1	0.1
**C7-G10**	7	0.6	0.1	-0.1	0.6	0.1	-0.1
**G8-C9**	8	-	-	-	-	-	-
	Base pair step parameter						
		**Slide (Å)**	**Shift (Å)**	**Rise (Å)**	**Roll (°)**	**Twist (°)**	**Tilt (°)**
**Base-pair step**	**Step identity**						
**$C_1G_2/C_{15}G_{16}$**	1	-	-	-	-	-	-
**$G_2T_3/A_{14}C_{15}$**	2	-0.3	-0.2	3.5	-0.29	32.0	-0.2
$T_3A_4/T_{13}A_{14}$	3	-0.1	0.2	3.2	-0.12	34.1	0.2
$A_4T_5/A_{12}T_{13}$	4	-0.5	0.4	3.1	-0.53	33.3	0.4
$T_5A_6/T_{11}A_{12}$	5	-0.6	0.2	3.5	-0.62	31.6	0.2
**$A_6C_7/G_{10}T_{11}$**	6	-0.2	0.4	3.3	-0.24	32.4	0.4
**C_7G_8/C_9G_{10}**	7	-	-	-	-	-	-

**(B)**	**DA4-DNA complex**						
	Base pair parameter						
		**Buckle (°)**	**Propeller (°)**	**Opening (°)**	**Shear (Å)**	**Stretch (Å)**	**Stagger (Å)**
**Base-pair**	**Base-pair identity**						
**C1-G16**	1	-2.6	-3.1	0.0	0.20	-0.2	0.05
**G2-C15**	2	-21.2	-3.9	-0.8	-0.30	-0.1	-0.2
T3-A14	3	-7.5	-5.9	5.3	-0.06	-0.1	0.1
A4-T13	4	-0.7	-11.5	4.6	-0.07	-0.2	-0.01
T5-A12	5	0.9	-11.6	4.5	0.06	-0.2	-0.03
A6-T11	6	8.7	-5.7	4.7	0.15	-0.08	0.04
**C7-G10**	7	18.0	-6	2.5	0.47	-0.1	0.02
**G8-C9**	8	3.2	-2.9	-0.5	-0.23	-0.2	0.09
	Base pair step parameter						

		Slide (Å)	Shift (Å)	Rise (Å)	Roll (°)	Twist (°)	Tilt (°)
**Base-pair step**	**Step identity**						
**$C_1G_2/C_{15}G_{16}$**	1	1.7	-2.1	7.0	-3.7	28.8	-12.1
**$G_2T_3/A_{14}C_{15}$**	2	0.06	0.3	3.0	5.8	24.2	-3.1
$T_3A_4/T_{13}A_{14}$	3	1.04	-0.4	3.3	8.7	37.3	-1.7
$A_4T_5/A_{12}T_{13}$	4	-0.5	-0.0	3.2	6.9	27.0	-0.07
$T_5A_6/T_{11}A_{12}$	5	1.0	0.4	3.3	8.5	37.6	2.4
**$A_6C_7/G_{10}T_{11}$**	6	0.05	-0.2	3.1	5.4	25.8	1.0
**C_7G_8/C_9G_{10}**	7	1.5	2.0	7.0	-4.0	26.7	14.0

**(c)**	**DA5-DNA complex**						
	Base pair parameter						
		**Buckle (°)**	**Propeller (°)**	**Opening (°)**	**Shear (Å)**	**Stretch (Å)**	**Stagger (Å)**
**Base-pair**	**Base-pair identity**						
**C1-G16**	1	1.4	3.2	-1.1	0.3	-0.4	-0.1
**G2-C15**	2	-14.8	-4.2	0.2	-0.3	-0.2	0.2
T3-A14	3	-8.6	-3.4	3.9	-0.1	-0.1	0.0
A4-T13	4	0.2	-12.5	2.8	-0.1	-0.2	0.1
T5-A12	5	6.6	-11.5	3.4	0.1	-0.1	-0.1
A6-T11	6	-1.1	-6.6	4.9	-0.0	-0.2	-0.1
**C7-G10**	7	16.5	6.4	-1.2	0.2	-0.1	-0.0
**G8-C9**	8	1.0	6.6	1.7	-0.3	-0.1	-0.2
	Base pair step parameter						
		**Slide (Å)**	**Shift (Å)**	**Rise (Å)**	**Roll (°)**	**Twist (°)**	**Tilt (°)**
**Base-pair step**	**Step identity**						
**$C_1G_2/C_{15}G_{16}$**	1	1.6	-2.3	7.1	-3.2	27.4	-14.0
**$G_2T_3/A_{14}C_{15}$**	2	0.2	0.2	3.3	6.0	26.5	-1.0
$T_3A_4/T_{13}A_{14}$	3	1.0	-0.6	3.2	6.2	38.1	-2.5
$A_4T_5/A_{12}T_{13}$	4	-0.4	0.2	3.2	7.1	26.4	1.2
$T_5A_6/T_{11}A_{12}$	5	0.7	1.0	3.5	3.5	40.0	3.5
**$A_6C_7/G_{10}T_{11}$**	6	0.1	0.6	3.0	10.0	20.6	3.8
**C_7G_8/C_9G_{10}**	7	1.4	2.5	6.9	-3.0	28.2	14.6

D. Supplementary References

1. Marenich, A.V., Cramer, C.J. and Truhlar, D.G. (2009) Universal Solvation Model Based on Solute Electron Density and on a Continuum Model of the Solvent Defined by the Bulk Dielectric Constant and Atomic Surface Tensions. *The Journal of Physical Chemistry B*, **113**, 6378-6396.
